# Lysophosphatidic Acid Is a Proinflammatory Stimulus of Renal Tubular Epithelial Cells

**DOI:** 10.3390/ijms23137452

**Published:** 2022-07-05

**Authors:** Christiana Magkrioti, Georgia Antonopoulou, Dionysios Fanidis, Vaia Pliaka, Theodore Sakellaropoulos, Leonidas G. Alexopoulos, Christoph Ullmer, Vassilis Aidinis

**Affiliations:** 1 Institute for Fundamental Biomedical Research, Biomedical Sciences Research Center Alexander Fleming, 16672 Athens, Greece; magkrioti@fleming.gr (C.M.); antonopoulou@fleming.gr (G.A.); fanidis@fleming.gr (D.F.); 2 ProtATonce Ltd., 15343 Athens, Greece; vicky.pliaka@protatonce.com (V.P.); teosakel@gmail.com (T.S.); leo@protatonce.com (L.G.A.); 3 School of Mechanical Engineering, National Technical University of Athens, 15780 Zografou, Greece; 4 Roche Pharmaceutical Research and Early Development, Roche Innovation Center Basel, F. Hoffmann-La Roche Ltd., 4070 Basel, Switzerland; christoph.ullmer@roche.com

**Keywords:** lysophosphatidic acid, inflammation, tubular epithelial cells, cytokines

## Abstract

Chronic kidney disease (CKD) refers to a spectrum of diseases defined by renal fibrosis, permanent alterations in kidney structure, and low glomerular-filtration rate. Prolonged epithelial-tubular damage involves a series of changes that eventually lead to CKD, highlighting the importance of tubular epithelial cells in this process. Lysophosphatidic acid (LPA) is a bioactive lipid that signals mainly through its six cognate LPA receptors and is implicated in several chronic inflammatory pathological conditions. In this report, we have stimulated human proximal tubular epithelial cells (HKC-8) with LPA and 175 other possibly pathological stimuli, and simultaneously detected the levels of 27 intracellular phosphoproteins and 32 extracellular secreted molecules with multiplex ELISA. This quantification revealed a large amount of information concerning the signaling and the physiology of HKC-8 cells that can be extrapolated to other proximal tubular epithelial cells. LPA responses clustered with pro-inflammatory stimuli such as TNF and IL-1, promoting the phosphorylation of important inflammatory signaling hubs, including CREB1, ERK1, JUN, IκΒα, and MEK1, as well as the secretion of inflammatory factors of clinical relevance, including CCL2, CCL3, CXCL10, ICAM1, IL-6, and IL-8, most of them shown for the first time in proximal tubular epithelial cells. The identified LPA-induced signal-transduction pathways, which were pharmacologically validated, and the secretion of the inflammatory factors offer novel insights into the possible role of LPA in CKD pathogenesis.

## 1. Introduction

Chronic kidney disease (CKD), with a worldwide prevalence of 13.4% [1], refers to a spectrum of diseases defined by permanent alterations in kidney structure or function. The most prominent pathological characteristic of CKD is renal fibrosis, while the gradual replacement of podocytes (in the glomeruli) and the tubulointerstitium with an extracellular matrix (ECM) leads to irreversible nephron loss [2,3]. Renal tubular epithelial cells (TECs) are the cells lining the nephrons, and the ones responsible for the selective transport of minerals, organic compounds, and water into and out of the tubular fluid of the nephrons, however, they are vulnerable to injuries. An injury can cause the loss of epithelial-cell polarization and intercellular contacts, epithelial to mesenchymal transition (EMT), cell death and, finally, abrogation of the tubular function [3]. The initial injury is followed by repair mechanisms and epithelial regeneration; but, depending on the severity and frequency of the initial injuries, these repair mechanisms may become maladaptive and the epithelial damage may progress to CKD [3,4,5]. Prolonged epithelial-tubular damage involves cell death, mitochondrial dysfunction, metabolic disturbance, oxidative stress, cell-cycle arrest and senescence, partial EMT, epigenomic modifications, and proinflammatory mediators’ secretion [3,5,6,7,8], with the latter further fueling the tubular injury [9]. Damaged TECs express CCL2/ MCP1 (monocyte chemoattractant protein-1) and CCL5/RANTES (regulated on activation, normal T cell expressed and secreted), thus mobilizing macrophages and dendritic cells to the site of the injury, a factor associated with CKD [10]. Macrophages, on their part, secrete a number of factors that further activate inflammation and fibrosis, although their functions vary depending on their polarization [8]. Furthermore, during the transition to CKD and the phenotypical changes of epithelial cells, the tubular basement membrane may be disrupted by activated matrix metalloproteinases allowing myofibroblast intrusion in the interstitium and, thus, promoting tubulointerstitial fibrosis and CKD progression [3]. All the above highlight the importance of TECs as initiators of tubulointerstitial fibrosis [8].

Apart from fibrosis, the progression of CKD has been shown to lead to inflammation and oxidative stress, suggesting that CKD is a low-grade inflammatory process [11,12]. Indeed, the lower GFR and the higher albuminuria of CKD patients are associated with higher levels of inflammatory cytokines, such as IL-1β and IL-6 in their plasma [13]. This upsurge is partly owed to the prolonged half-life of cytokines due to the impaired excretory renal function but also to increased tissue production. Subsequently, this amplified chronic inflammation may lead to high mortality in CKD patients [13].

Lysophosphatidic acid (LPA) is a lysophospholipid present in most biological fluids. LPA is actually a mixture of species carrying various saturated or unsaturated fatty acids. LPA presents many functions due to its signaling through at least six cognate receptors (LPAR1-6), which are further coupled with G proteins, activating numerous signal-transduction pathways [14]. LPA signaling is implicated in several chronic inflammatory or fibrotic diseases, such as rheumatoid arthritis (RA), cardiovascular diseases, pulmonary and liver fibrosis, and others [14]. Additionally, LPA and its receptors are also involved in CKD. As early as 1998, LPA levels in the plasma of patients with renal failure on hemodialysis were found to be higher compared to healthy controls [15]. Two LPA species (16:0 and 18:2) were among a panel of seven metabolites that discriminated the sera of patients with CKD of diverse aetiologies from the sera of healthy subjects [16]. Moreover, LPA 16:0 and LPA 20:4 were found to have risen in the urine of type II diabetes patients with nephropathy compared to type II diabetes patients without nephropathy, probably due to higher local production [17]. Furthermore, the LPA-producing enzyme, Autotaxin (ATX), and LPAR1 and LPAR3 were found to have increased in diabetic human kidneys compared to healthy kidneys [18]. Concerning TECs, LPA promotes the expression or activation of profibrotic molecules [19]. Specifically, LPA induces integrin αvβ6-mediated latent transforming growth factor beta (TGFβ) activation, which leads to the increase in connective tissue growth factor (CTGF) and platelet-derived growth factor (PDGFB) mRNA and protein expression in a TGFβ-dependent fashion in several TEC cell lines and primary cultures [19]. Additionally, LPA treatment on mouse renal TECs induces TGFβ mRNA expression [20,21]. The above conclude that LPA has an effect in the secretory pattern of fibrosis-related factors from TECs. 

In an effort to expand our knowledge on TECs’ signaling and secretion of proinflammatory/profibrotic factors, we exposed the kidney proximal tubular epithelial cell line HKC-8, which derives from the normal kidney cortex [22], to 175 inflammatory-immunological stimuli and measured the levels of 27 intracellular phosphoproteins as well as 32 extracellular secreted mediators upon each one of the stimuli employing custom multiplex ELISA. Furthermore, we exposed HKC-8 to three different species of LPA in order to investigate the effect of LPA on TECs. According to our findings, LPA induced the phosphorylation of CREB1, ERK1, IκΒα, JUN, and MEK1 and the secretion of proinflammatory molecules CCL2, CCL3, CXCL10, ICAM1, IL-6, and IL-8.

## 2. Results

### 2.1. Exposure of HKC-8 Cells to LPA and 175 Other Stimuli

**Figure 1:**
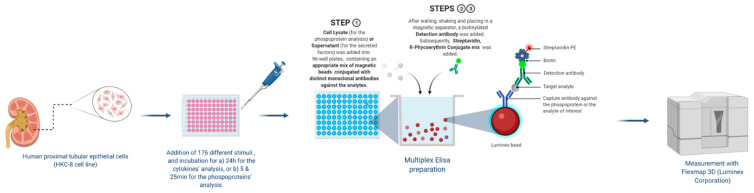
Multiplex ELISA for the detection of secreted factors and signaling molecules phosphorylation. Human kidney proximal tubular epithelial cells, HKC-8, were stimulated with 176 stimuli. Supernatants were collected at 24 h and cell lysates at 5 and 25 min post stimulation. Supernatants or cell lysates were added to a mix containing magnetic beads internally dyed with precise proportions of red and infrared fluorophores, thus, rendering unique spectral signature microspheres. Each unique microsphere-bead was conjugated with a distinct monoclonal antibody against a secreted factor or a phosphoprotein. Biotinylated detection antibodies were added to the mix, followed by a streptavidin-R-Phycoerythrin complex. This process allows the simultaneous recognition of 32 secreted factors or 27 phosphoproteins in one sample. Created with BioRender.com, accessed on 1 June 2022.

**Figure 2:**
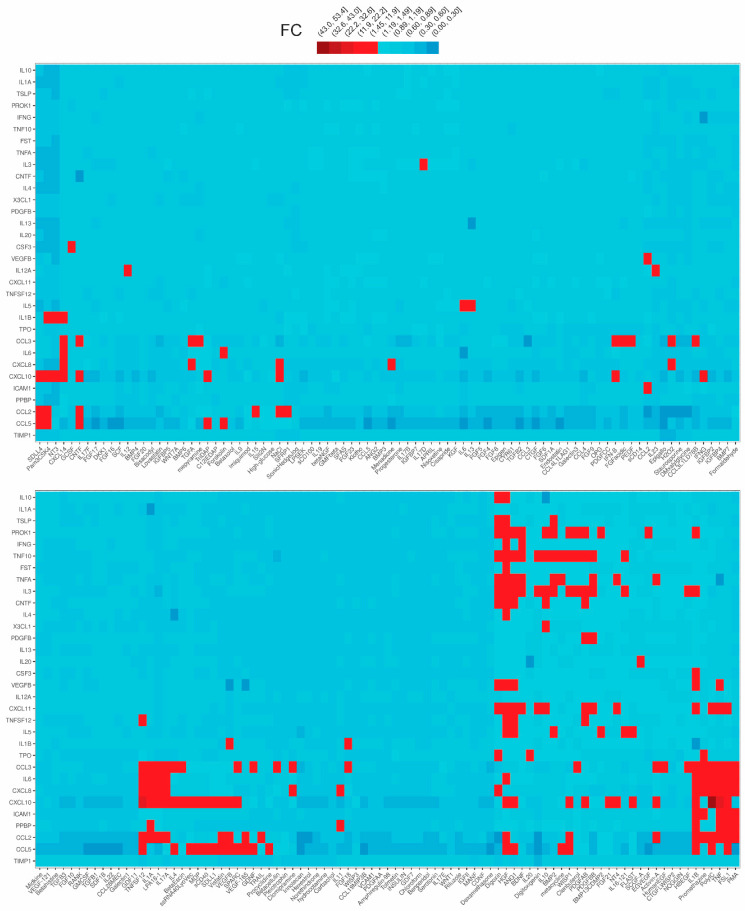
Differential expression of 32 secreted biological factors in the supernatants of human kidney proximal tubular epithelial cells (HKC-8) upon the stimulation with LPA (18:1) and 175 disparate biological stimuli. The expression was assessed with multiplex ELISA employing microbeads of unique spectral signatures conjugated with monoclonal antibodies specific for each of the 32 secreted factors. Red indicates active signals (FC ≥ 1.5). See also Figure S1.

**Figure 3:**
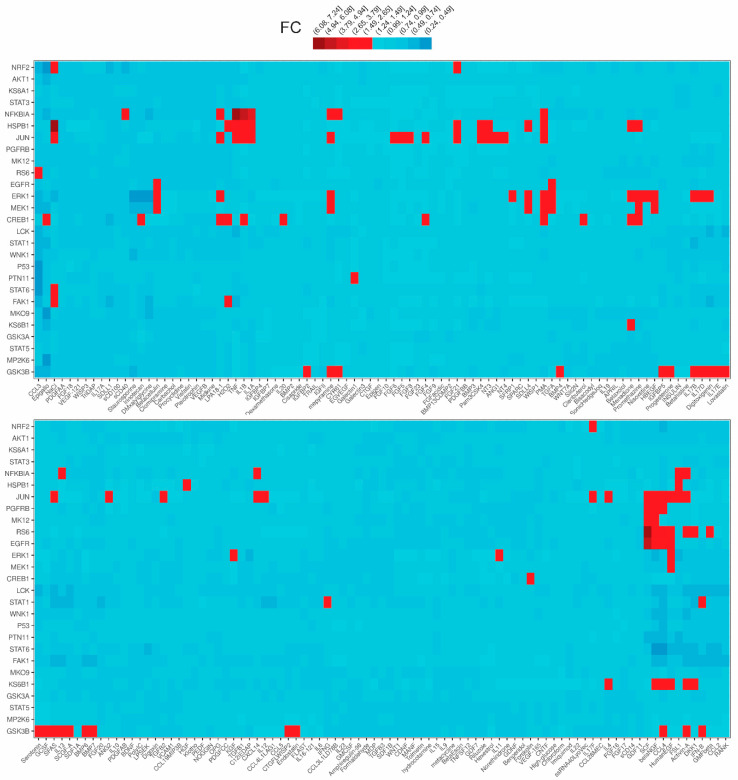
Phosphorylation of 27 major hubs in intracellular signaling pathways of human kidney proximal tubular epithelial cells (HKC-8) upon the stimulation with LPA (18:1) and 175 disparate biological stimuli. The expression was assessed with multiplex ELISA employing microbeads of unique spectral signatures conjugated with monoclonal antibodies specific for each of the 27 phosphoproteins. Red indicates active signals (FC ≥ 1.5). See also Figure S1.

In an attempt to shed light on the signaling of TECs, we employed the human renal proximal tubular epithelial cell line HKC-8 and exposed it to LPA and 175 other inflammatory-immunological stimuli. Stimuli comprised toll-like receptor (TLR) ligands, cytokines, chemokines, growth factors and drugs (Tables S1 and S2). The experimentation included two sub-experiments. In the first sub-experiment, cells were exposed to the stimuli for 24 h and supernatants were collected, while in the second sub-experiment the exposure lasted for 5 or 25 min and cell lysates were collected. Subsequently, we measured the levels of 32 extracellular secreted factors in the supernatants and the levels of 27 intracellular phosphoproteins in the lysates upon each one of the stimuli employing custom multiplex ELISA. This method is based on the usage of magnetic beads of unique spectral signatures, conjugated with antibodies against each of the analytes. Recognition is achieved with biotinylated detection antibodies and streptavidin conjugated with R-Phycoerythrin (Figure 1). For each stimulus, the levels of the analytes at the stimulated state were divided with the levels of the analytes at the unstimulated state (median of the control wells) and the emerging ratios (fold-changes, FCs) correspond to the normalized responses. The responses to the different stimuli are depicted as FCs in the heatmap plots of Figure 2 and Figure 3. These plots show in detail the activations (or not) of each analyte upon each stimulus. The color code indicates the level of response, with light blue referring to low or no response and dark red referring to a high response/ activation. A response was considered active when the expression of an analyte upon a stimulus was higher than 1.5 FCs, which was set as the threshold in our analysis. The selection of the threshold was made based on the sensitivity analysis of Supplementary Figure S1, where it can be seen that at a threshold equal to 1.5, the number of activations is rather stable and also high enough to allow for the subsequent analysis. Regarding the phosphoproteins’ sub-experiment, which was done at two time points (5′ and 25′), the heatmap depicts an active response when the FC is above the threshold at at least one time point.

Among the 176 stimuli tested, 108 were the active ones at either sub-experiment. In total, 76 stimuli gave an active response in the secreted factors sub-experiment (Figure 2) and 71 in the phosphoproteins sub-experiment (Figure 3). Collectively, hepatocyte growth factor (HGF) was the stimulus that evoked the most active responses (18 secreted factors, 0 phosphoproteins), followed by interleukin 1β (13 secreted factors, 4 phosphoproteins), the synthetic TLR2/TLR6 ligand FSL1 (10 secreted factors, 3 phosphoproteins), Tumor-necrosis factor alpha or TNFα (9 secreted factors, 3 phosphoproteins), phorbol 12-myristate 13-acetate or PMA (6 secreted factors, 6 phosphoproteins), angiopoietin or ANG1 (11 secreted factors), digoxin (11 secreted factors), and others. Among the secreted factors, chemokine CXCL10 was the most common active responder (34 hits), followed by CCL3 (30 hits), while, among the signaling phosphoproteins, JUN was the molecule with the most activations (29 hits).

### 2.2. LPA Is a Proinflammatory Stimulus

**Figure 4:**
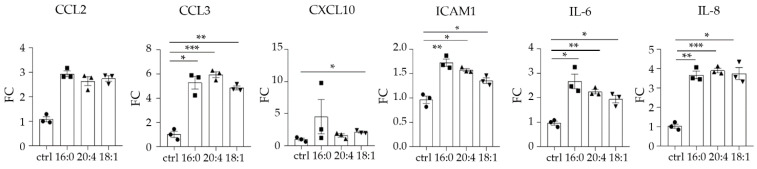
LPA stimulates the secretion of CCL2, CCL3, CXCL10, ICAM1, IL-6, and IL-8 from human kidney proximal tubular epithelial cells (HKC-8). Multiplex ELISA quantifying the expression of the indicated secreted factors in the supernatants from HKC-8 cells upon the stimulation with three different LPA species (16:0, 20:4, 18:1) at 10 μM for 24 h. Statistical significance was assessed with Brown–Forsythe’s and Welch’s ANOVA followed by Dunnett’s post hoc test in the case of normal distribution or with Kruskal–Wallis test in the case of non-normal distribution; * *p* < 0.05, ** *p* < 0.01, *** *p* < 0.001. Circles correspond to control values, squares correspond to LPA 16:0 values, upward triangles correspond to LPA 20:4 values and downward triangles correspond to LPA 18:1 values.

**Figure 5:**
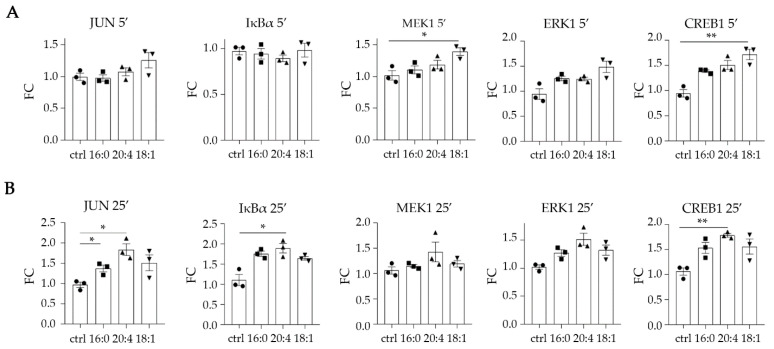
LPA stimulates the phosphorylation of JUN, IκΒα, MEK1, ERK1, and CREB1 in human kidney proximal tubular epithelial cells (HKC-8). Cells were incubated with three different LPA species (16:0, 20:4, 18:1) at 10 μΜ for 5 (**A**) or 25 min (**B**), and the phosphorylation was assessed with multiplex ELISA in triplicates. Circles correspond to control values, squares correspond to LPA 16:0 values, upward triangles correspond to LPA 20:4 values and downward triangles correspond to LPA 18:1 values. Statistical significance was assessed with Brown–Forsythe’s and Welch’s ANOVA followed by Dunnett’s post hoc test in the case of normal distribution or with Kruskal–Wallis test in the case of non-normal distribution. * *p* < 0.05, ** *p* < 0.01.

One of the 176 stimuli to which HKC-8 cells were exposed was LPA 18:1, which led to several activations. Concerning the secreted factors, it induced the secretion of interleukin 6 (IL-6), interleukin 8 (IL-8), and chemokines CCL2, CCL3, and CXCL10 (Figure 2). With regard to the phosphoproteins, LPA 18:1 induced the phosphorylation of JUN, IκBα, ERK1, and CREB1 (Figure 3). Subsequently, in an effort to verify the LPA results, we performed a second experiment using two more LPA species, LPA 16:0 and 20:4, on top of LPA 18:1. This experiment was done in triplicates, thus allowing statistical analysis. With a threshold of FC ≥ 1.5, a number of active signals was detected upon the three LPA species (Figure 4 and Figure 5). Most of the responses were shared between them, but some differences exist, which, however, need further exploration. Regarding the secreted factors, CCL3, IL-6, and IL-8 were expressed statistically significantly higher than the medium upon all three species (Figure 4). CCL2 was expressed at levels higher than the medium upon them as well, however, not statistically significantly. Soluble Intercellular Adhesion Molecule 1 (sICAM1) was also induced upon all three species statistically significantly, however, only upon LPA 16:0 and 20:4 above the FC = 1.5 (Figure 4). LPA 18:1 also triggered the expression of CXCL10 statistically significantly.

Pertaining to the signaling phosphorylations, components that were phosphorylated above the threshold upon the three LPA species were IKBα (25′) and CREB1 (25′), with a statistical significance for LPA 20:4 (Figure 5). JUN (25′) was phosphorylated by all three species with statistically significant phosphorylation upon LPA 16:0 (although below the FC = 1.5) and 20:4. JUN (25′) gave an active signal upon LPA 18:1 too, albeit not statistically significantly. Moreover, LPA 18:1 statistically significantly induced the phosphorylation of MEK1 (5′) and CREB1 (5′) (Figure 5). Finally, ERK1 (5′) was phosphorylated upon LPA 18:1 and ERK1 (25′) upon LPA 20:4, but not statistically significantly.

### 2.3. Clustering and Responses of Other Stimuli

**Figure 6:**
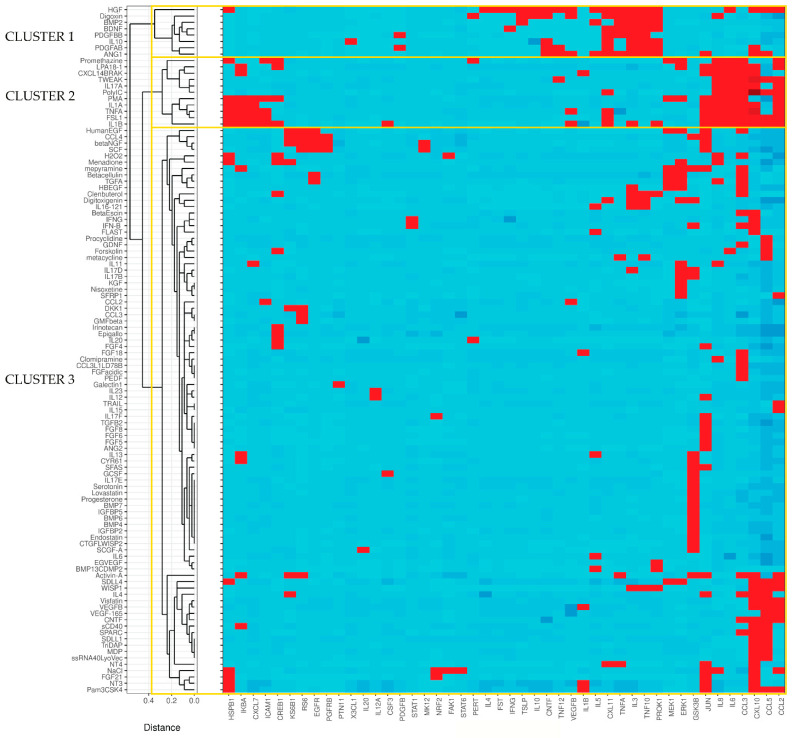
**LPA clusters with proinflammatory stimuli.** Heatmap of the active stimuli clustered in three major groups (1-3). Inactive stimuli and globally unresponsive signals were removed. Pairwise stimuli distance was calculated on binary transformed fold change values using Gower’s metric prior to divisive clustering. See also Figures S2–S4.

Trying to identify stimuli that affect the same kidney processes, we initially transformed absolute Luminex data into fold-change values, with respect to the median value of medium-treated cells, separately for secreted factors (Figure 2) and phosphoproteins plates (Figure 3). Subsequently, we concatenated all plates and focused on 108 stimuli that caused at least one activation event (activation threshold FC ≥ 1.5). After removing non-responsive signals, 46 signals remained. Their respective values were binary transformed (1: activation; 0: non-activation) and used for stimuli unsupervised clustering (Gower’s metric; divisive clustering), which led to the definition of three clusters (Figure 6).

As shown in Figure 6, LPA falls into the same cluster (cluster 2) as PMA, which is a PKC activator and a T-cell activator; IL-1α, IL-1β, IL-17α, and TNFα, which are proinflammatory cytokines; FSL1, a lipoprotein derived from *Mycoplasma salivarium* and a TLR ligand; PolyIC, a synthetic analogue of double-stranded RNA and a TLR3 ligand; promethazine, an antihistamine drug; TNFSF12/TWEAK, which is a TNF family member; and CXCL14/BRAK that is a breast and kidney-expressed chemokine activating B cells and monocytes. Between LPA and the aforementioned molecules of its cluster, many similarities appear in the secreted factors pattern induction; IL-6, IL-8 and CCL3 are induced by all 11 members of the cluster, while CCL2 and CXCL10 are induced by most of them. The results of the phosphoproteins also indicate the signaling convergence between LPA and the other molecules of cluster 2, as most of them induce the phosphorylation of JUN and IκBα. Promethazine, PMA and IL-1β also lead to the phosphorylation of CRΕΒ1, like LPA does. 

Another cluster that emerges is the cluster 1, which includes angiopoietin 1 (ANG1), HGF, platelet-derived growth factor AB (PDGFAB), PDGFBB, digoxin, bone morphogenetic protein 2 (BMP2), brain-derived neurotrophic factor (BDNF), and IL-10. These stimuli induce a series of secreted cytokines such as Prokineticin 1 (PROK1), Ciliary Neurotrophic Factor (CNTF), TNF family members, IL-3, CXCL11, and others. ANG1, which triggered the secretion of several factors (IL-3, TNFα, TNF10, PROK1, CNTF, TNF12, VEGFB, CXL11, IL-5, CXCL10, CCL5, IL-3), is thought to be protective in models of renal injury [23], while BMP2, which induced TSLP, IL-3, TNFα, TNF10, PROK1, and IL-5, has been found to induce the commitment of adult renal progenitor cells (ARPCs) toward a myofibroblastic phenotype [24]. 

The rest of the 108 active stimuli, out of the 176 used in our experiments, form cluster 3. Here, we are mentioning some of these stimuli that have also been linked with renal fibrosis. For example, the proinflammatory cysteine-rich protein 61 (Cyr61), which is increased in a mouse model of renal fibrosis [25], induced the phosphorylation of IκBα and GSK3B in our experimental setup. Activin A, which activates renal interstitial fibroblasts during the fibrotic processes of the kidney [26], also triggered the phosphorylation of many intracellular components (GSK3B, KS6B1, RS6, JUN, IκBα) and the secretion of chemokines CCL3, CXCL10, and CCL2 in HKC-8 cells. WNT1-inducible-signaling pathway protein 1 (WISP1), which has been found to regulate kidney fibrosis through TGFβ [27] and whose serum levels are elevated in CKD patients [28], promoted the secretion of many factors too (CCL2, CCL5, CXCL10, TNF10, PROK1, IL-3). It would, therefore, be interesting to attempt to find a connection between some of the observed responses and renal pathology.

### 2.4. Pathway-Enrichment Analysis, Microarray, and Single-Cell Analysis in Relation to Cluster 2

To better characterize cluster 2, we performed pathway-enrichment analysis against both GO and KEGG libraries (see Section 4). For this purpose, we maintained the gene-coded stimuli triggering at least one response, as well as the signals responding to at least one stimulus (Supplementary Figure S2A). Top enriched terms of all GO categories and KEGG database suggest that cluster 2 is functionally related with inflammatory processes and responses to pathogen invasion (Supplementary Figure S2B,C).

Next, to prioritize some of the cluster 2 features, we re-analyzed two microarray datasets containing control as well as CKD, focal segmental glomerulosclerosis, and immunoglobulin A nephropathy samples. The differentially expressed genes identified (pathology vs. steady-state condition) include genes encoding four cluster 2-induced molecules: TNFSF12, IL-1β, ICAM1, and CCL5 (Supplementary Figure S3). In fact, ICAM1 is upregulated upon kidney disease in all three groups of nephropathy samples, underscoring its importance in the context of kidney disease. Interestingly, according to our results, ICAM1 is activated, among others, by LPA, indicating potentially common mechanisms between LPA effects and kidney pathologies.

To examine cluster 2 features’ cell specificity, we have re-analyzed a single-cell RNA-seq dataset with kidney samples from CKD and control individuals [29]. Five cytokine genes induced by cluster 2 molecules were identified as marker genes of CD10^−^ proximal tubule epithelial cells (PT-neg), *CCL2, CCL3, CCL5, CXCL8* (the gene encoding IL-8), and *IL-1β*, while *VEGFB* was marking CD10^+^ proximal tubule epithelial cells (PT-pos) (Supplementary Figure S4A). Marker features did not overlap between the two PT clusters. The aforementioned results suggest that PT cells can indeed in vivo produce cluster 2-induced cytokines, several of them being responsive to LPA treatment (CCL2, CCL3, IL-8). Last, we performed a ligand-receptor (cell-to-cell) analysis, so as to identify the final recipient cells of these secreted cytokines. The SingleCellSignalR ligand-receptor database was enriched with specific interactions from CellTalkDB. All cells were grouped per population (epithelial, endothelial, mesenchymal, neuronal, immune) with the exception of the two PT cells’ sub-clusters, which remained intact. Focus on PT-marker cytokines suggests that PT cells could establish both autocrine as well as paracrine communications with all other cell populations, such as epithelial, mesenchymal, and endothelial cells, through specific ligand–receptor interactions (Supplementary Figure S4B).

### 2.5. LPA Induces the Expression of the Secreted Factors CCL2, CCL3, CXCL10, ICAM1, IL-6, and IL-8 at the mRNA Level in HKC-8 Cells

**Figure 7:**
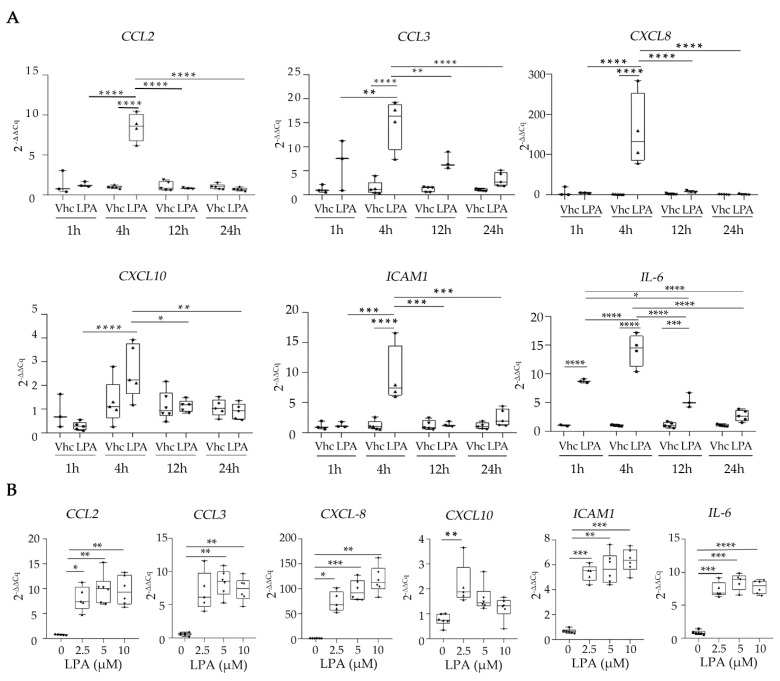
LPA stimulates the expression of *CCL2*, *CCL3*, *CXCL8*, *CXCL10*, *ICAM1*, and *IL6* from human kidney proximal tubular epithelial cells (HKC-8). (**A**,**B**) HKC-8 cells were incubated for 1, 4, 12, and 24 h with 10 μM of LPA (**A**), and with 2.5, 5, and 10 μM LPA for 4 h (**B**). Control cells were stimulated with the equivalent volume of chloroform (VHC). mRNA-expression levels of the indicated secreted factors were quantified with RT-qPCR. The Cq values of each gene were normalized against the Cq values of *B2M*. The results represent the findings of two (**A**) and three (**B**) separate experiments. In (**A**) circles, upward triangles, downward triangles and diamonds refer to 1, 4, 12 and 24 hours of incubation with LPA, respectively. In (**B**) circles, upward triangles, downward triangles and diamonds refer to incubation with 0, 2.5, 5 and 10 μM LPA, respectively. Statistical significance was assessed in (**A**) with 2-way ANOVA and Tukey’s post hoc test and in (**B**) with Brown-Forsythe’s and Welch’s test or the Kruskal–Wallis test depending on the normality status of the data; * *p* < 0.05, ** *p* < 0.01, *** *p* < 0.001, **** *p* < 0.0001. See also Figure S5.

In order to verify the expression of the observed secreted factors upon LPA, we studied their mRNA expression. To this end, we stimulated HKC-8 cells with LPA 18:1, collected the cells, and completed the RNA extraction. Initially, we stimulated them for different time periods (0, 1, 4, 12, and 24 h). According to Figure 7A, all the analyzed secreted molecules were maximally induced at 4 h; therefore, we chose this time point for our subsequent experiments. Next, we stimulated HKC-8 cells with different concentrations of LPA (0, 2.5, 5, and 10 μM) for 4 h and observed a dose-dependent induction of the indicated molecules with a maximum effect at 10 μM (Figure 7B). Both the time-dependent and dose-dependent experiments verified a strong induction of *CCL2*, *CCL3*, *CXCL8*, *ICAM1*, and *IL-6* by LPA 18:1, with *CXCL8* transcription being activated over a hundred times. *CXCL10* was only marginally induced by LPA at a concentration of 2.5 μM. Finally, we analyzed the expression of LPARs in HKC-8 cells and found that *LPAR2* is the highest expressed LPAR in these cells, followed by *LPAR6*, with no significant alterations of the LPARs pattern upon LPA stimulation (Supplementary Figure S5).

### 2.6. LPA-Induced Cellular Signaling Pathways

**Figure 8:**
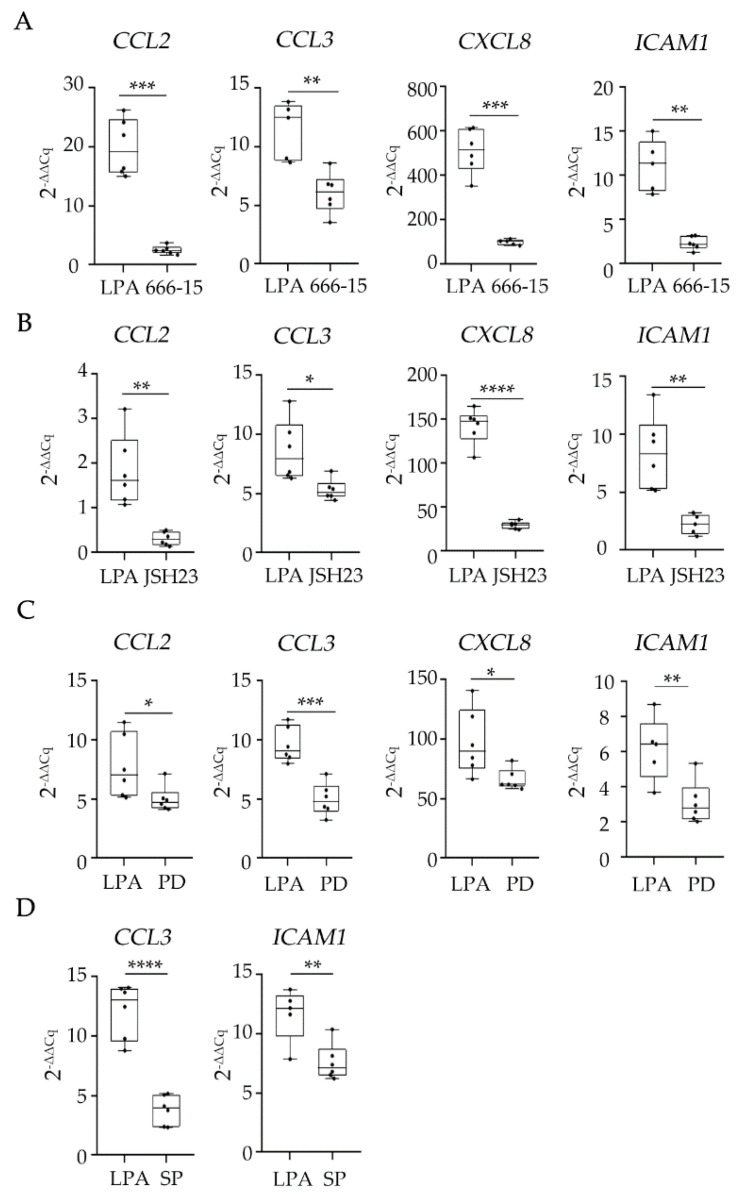
Pharmacologic dissection of LPA-induced cellular signaling pathways. HKC-8 cells were pretreated for 1 h with 666-15 (CREB1 inhibitor) 10 μM in (**A**), JSH23 (NFκΒ inhibitor) 100 μM in (**B**), PD98059 (MEK/ERK inhibitor) 50 μM in (**C**), or SP600125 (JNK inhibitor) 50 μM in (**D**) and then activated with LPA at a final concentration of 10 μΜ for 4 h. mRNA-expression levels of the indicated secreted factors were quantified with RT-qPCR. The Cq values of each gene were normalized against the Cq values of *B2M*. Statistical analysis was performed with unpaired *t*-test or Welch’s test in the case of normal data and with Mann–Whitney in the case of non-normal data. * *p* < 0.05, ** *p* < 0.01, *** *p* < 0.001, **** *p* < 0.0001. See also Figure S6.

**Figure 9:**
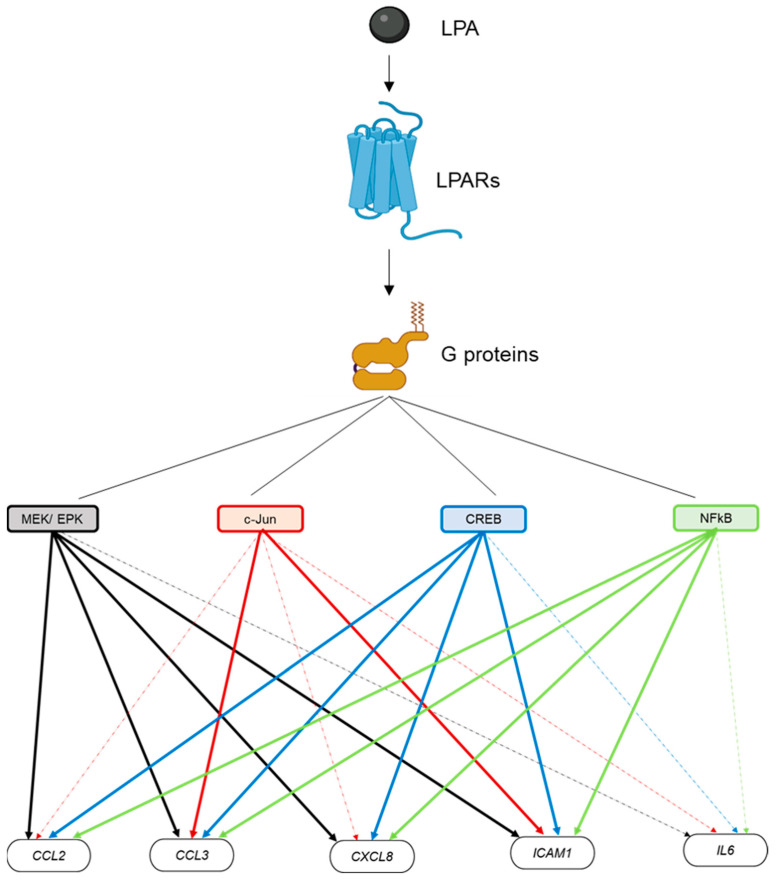
Graphical depiction of the LPA-induced signaling pathways in HKC-8 cells. LPA binds to the LPARs, which activate G proteins and the signal progresses to secondary signaling hubs, such as MEK/ERK or transcription factors c-JUN, CREB1, and NFκB. MEK/ERK, CREB1, and NFκB co-activate *CCL2*, *CCL3*, *CXCL8* (IL-8), and *ICAM1* expression. C-JUN activates only *CCL3* and *ICAM1* expression. Solid colored lines show connections that are derived from our results. Connections depicted with dashed lines are drawn from the literature and are not verified from our data. Created with BioRender.com, accessed on 1 June 2022.

In order to elucidate which LPARs and signaling pathways are implicated in the LPA-mediated induction of the secreted factors, we employed several LPARs and signaling pathway inhibitors (based on the proteins that were found phosphorylated upon LPA) and checked the expression of the secreted factors, apart from the low-expressed CXCL10, in their presence. We identified LPAR1/3 as the receptors responsible for the LPA-induced *CCL3*, *CXCL8*, and *IL-6* transcription, while LPAR2 was responsible for the *IL-6* transcription as well (Supplementary Figure S6). In the absence of inhibitors for other LPARs, we cannot exclude that signaling through LPAR6 or the other LPARs is participating in the regulation of the LPA-induced secreted factors. Regarding the phosphoproteins, we found that SP600125, a c-Jun N-terminal kinase (JNK) inhibitor, inhibits the expression of *CCL3* and *ICAM1* (Figure 8). Furthermore, in the presence of PD98059, a MEK/ERK inhibitor; JSH23, an NFκB inhibitor; and 666-15, a Creb inhibitor, *CCL2*, *CCL3*, *CXCL8*, and *ICAM1* expression was diminished (Figure 8). Our results suggest that the JNK/c-JUN, MEK/ERK, NFκB, and CREB pathways are implicated in the induction of some of the LPA-mediated secreted factors. In fact, *CCL2*, *CCL3*, *CXCL8,* and *ICAM1* are coregulated by these pathways simultaneously, as shown in Figure 9.

## 3. Discussion

In this study, we have performed custom multiplex ELISA in HKC-8 cells, a cell line of proximal tubular epithelial cells, which in vivo constitute an abundant cell population of the kidney. We have stimulated these cells with LPA and 175 immunological stimuli and monitored the phosphorylations of signaling molecules and the secretion of immune-related factors, such as cytokines, chemokines, and adhesion molecules. Out of the 176 stimuli, 108 were the active ones, with HGF evoking the most responses. Chemokine CXCL10 was the most common active responder among the secreted factors, while JUN was the signaling molecule with the most activations. Regarding LPA, which is our molecule of interest, it induced the phosphorylation of JUN, IκBa, MEK1, CREB1, and, marginally, ERK1 and the secretion of CCL2, CCL3, CXCL10, ICAM1, IL-6, and IL-8, as observed with the usage of three different LPA species. In the context of verifying the LPA-induced expression of the six secreted factors at the mRNA level, we investigated the levels of the various LPARs in the HKC-8 cell line and found that the most prominent receptor is LPAR2, followed by LPAR6. The prevalence of LPAR2 in HKC-8 cells is corroborated by other investigations studying the presence of LPARs in mouse renal TEC cell lines [19,20,21]. Subsequently, we verified the LPA-induced transcription of *CCL2*, *CCL3*, *CXCL8* (the gene encoding IL-8), *ICAM1*, *IL-6*, and, marginally, *CXCL10* in these cells. 

TECs are known sources of CCL chemokines [30]. These molecules have a critical role in progressive renal injury, as suggested by functional blocking studies, including treatment with neutralizing antibodies to CCL chemokines or their receptors, truncated chemokines, or small molecule-receptor antagonists [30]. They are important regulators of leukocyte recruitment during renal injury. Both CCL2, also known as monocyte chemoattractant protein 1 (MCP-1), and CCL3, also known as macrophage inflammatory protein-1α (MIP-1α), which are induced by LPA, form gradients that drive infiltration of monocytes/macrophages, T cells, and B cells to the sites of injury [31]. More specifically, CCL2 is released by TECs after renal injury, inducing the influx of CCL2 receptor, CCR2-positive cells such as monocytes, dendritic cells, T cells, and fibrocytes [31]. Monocytes differentiate in M1/M2 macrophages, with M1 producing proinflammatory cytokines, such as TNFα, IL-1β, IL-6 and CCL2 and with M2 promoting wound healing and leading to TGF-β and anti-inflammatory cytokines’ expression [31]. CCL2 is implicated in the pathogenesis of several diseases with a strong monocytic component. In the context of renal pathophysiology, CCL2 participates in glomerulonephritis (GN) [32,33], DN [34,35], and the CKD model of unilateral ureter obstruction (UUO) [36]. Chemical or genetic ablation of CCR2 reduces renal fibrosis, TGFβ production, and macrophage accumulation in several models of CKD [31]. Additionally, antagonism of CCR2 has positive effects in patients with type 2 DN [37]. LPA has been found to induce CCL2 production in mesangial cells and proximal tubular epithelial cells HK2 in vitro [38,39], corroborating the ability of LPA to promote CCL2 expression. 

Regarding CCL3, this is a chemokine involved in the acute inflammatory state in the recruitment and activation of polymorphonuclear leukocytes. Elevated levels of CCL3 and its receptors CCR1 and CCR5 have been found upon UUO [36]. A chemical blockade of CCR1 reduces inflammation and interstitial fibrosis in CKD murine models, such as adriamycin-induced nephropathy and UUO [40,41]. To our knowledge, the induction of CCL3 by LPA has not been shown before.

CXCL10, also known as the 10-kDa interferon-inducible protein (IP-10), is a proinflammatory chemokine, as it is involved in the chemoattraction of monocytes, macrophages, T cells, and natural killer (NK) cells [42]. High CXCL10 levels have been detected in kidney biopsy specimens from patients with mesangial proliferative GN, where CXCL10 can directly contribute to mesangial cell proliferation [43]. CXCL10 levels are increased in the course of the UUO model [36]. *Cxcl10^−/−^* mice exhibit decreased proliferation with less ECM deposition and fewer cells in the glomeruli compared to wild-type mice [43]. On the contrary, recombinant murine CXCL10 reduces many indices of CKD in diabetic mice [44] and blocking CXCL10 promotes progressive renal fibrosis [45]. Therefore, more studies are needed to elucidate the role of CXCL10 in chronic renal disease.

ICAM1 or CD54 are also amplified by LPA in our experimental setup. LPA has been shown before to induce ICAM1 expression in epithelial ovarian cancer cells [46]. ICAM1 is a cell-surface glycoprotein that binds to integrins and participates in intercellular communication. Typically, it is expressed on endothelial and immune-system cells. ICAM1 is a ligand of lymphocyte-function-associated antigen-1 (LFA-1), which is a member of the integrin family found on leukocytes [47]. Via the ICAM1/LFA-1 interaction, ICAM1 stabilizes cell–cell interactions and facilitates the endothelial transmigration of leukocytes from the circulation to the sites of inflammation. ICAM1 is not detected in the TECs of healthy kidneys, whereas it is expressed in these cells upon GN [48]. Similarly, it is expressed in primary glomerulosclerosis compared to healthy renal regions, which do not express it [49]. Accordingly, the tubulointerstitial expression of ICAM1 has been suggested as a marker of injury in IgA nephropathy [50]. The levels of serum ICAM1 are increased in diabetes and its expression has been associated with DN [51]. Based on our analysis, ICAM1 is found upregulated in microarrays from kidneys of patients with CKD, focal segmental glomerulosclerosis, and IgA nephropathy compared to controls, thus underscoring its importance in all these situations. Other cluster 2-induced molecules that are found upregulated in these microarrays are TNFSF12/TWEAK, CCL5, and IL-1β, however, only ICAM1 is induced by LPA.

IL-6 is an interleukin that acts both as a proinflammatory cytokine and an anti-inflammatory myokine. When secreted by T cells and macrophages, it stimulates immune responses that lead to inflammation, e.g., during infection or trauma [52]. IL-6 signaling promotes T cell proliferation and apoptosis resistance; it is implicated in CD4^+^ T cell differentiation and plays a key role in the T-cell-mediated immune response, whereas it is indirectly involved in B cell-induced inflammation [53]. Therefore, IL-6 stimulates the inflammatory and auto-immune processes in many diseases. In the context of the kidney, the serum levels of IL-6 are significantly higher in CKD patients compared to healthy subjects [54] and numerous kidney resident cells, such as endothelial cells, mesangial cells, podocytes, and TECs can secrete it [53]. Several stimuli, such as glomerular injury, can induce IL-6 production from renal TECs, thus promoting a TEC–glomeruli crosstalk [53]. LPA has been shown before to induce IL-6 production in human bronchial epithelial cells [55], keratinocytes [56], and mesangial cells [38]. Exposure of mesangial cells to IL-6 and its soluble receptor (sIL-6R) together promotes the synthesis and secretion of CCL2/MCP1 and subsequently enhances monocyte recruitment [57]. Furthermore, IL-6 is implicated in fibrosis, as it can stimulate collagen I expression from TECs in vitro, while chronic administration of IL-6 enhances ischemia-reperfusion-induced fibrosis in vivo [58]. Interestingly, the blockade of IL-6 trans-signaling attenuates renal fibrosis and inflammation in the UUO model of kidney fibrosis [59]. 

IL-8, which is the molecule most prominently induced by LPA at the mRNA level, is a key mediator associated with inflammation as it causes the activation and chemotaxis of neutrophils, leading them towards the site of inflammation [60]. LPA has been shown to induce IL-8 production before, in the bronchial epithelial cells of the lungs [55,61,62], keratinocytes [56], and epithelial ovarian cancer cells [46]. In the kidney, human renal cortical epithelial cells express IL-8 upon incubation with IL-1β, TNF, or LPS [63]. Moreover, proximal and distal TECs are strongly positive for IL-8 in renal biopsies from patients with acute allograft rejection [63], and serum IL-8 levels are exacerbated in children with CKD [64]. In the kidneys of patients with T2 diabetes (T2D), glomerular IL-8 expression has been found to increase compared to controls [65]. Moreover, blockade of the IL-8-CXCR1/2 axis decreases diabetic-kidney-disease progression in mice [65]. 

Apart from CCL2, the rest of the secreted factors that we describe are shown for the first time to be LPA-induced in proximal TECs, and this may have an impact on several kidney pathologies. Given that all of the aforementioned molecules are proinflammatory, the LPA-induced secretome from TECs is characterized as proinflammatory and perhaps senescent too; five out of the six secreted factors are senescence-associated secretory phenotype proteins (SASP) [66,67]. Furthermore, the clustering of LPA with proinflammatory stimuli such as PMA, IL-1α, IL-1β, TNFα, IL-17α, TWEAK/TNFSF12, and FSL1 further enhances its proinflammatory characterization. Therefore, LPA is suggested to participate in the pathology of CKD.

With regard to the phosphorylated signaling proteins, LPA promoted phosphorylation of JUN, IκBA, CREB1, and, marginally, MEK1 and ERK. MEK1 is a signaling kinase upstream of ERK, which is an extracellular signal-regulated kinase that has been shown before to be phosphorylated in the presence of LPA in lung epithelial cells [61,68]. The three other proteins activated by LPA are transcription factors. JUN, in combination with FOS, forms the AP-1 early-response transcription factor. It is activated through double phosphorylation by the JNK pathway and is involved in cell-cycle progression and cancer. AP-1 transcribes numerous genes related to the inflammatory response, including cytokines (e.g., *TNFα*), chemokines (e.g., *CCL2*), and leukocyte-adhesion molecules (e.g., *VCAM-1*) [69]. A study has illustrated before an LPA-induced phosphorylation of JUN in human bronchial epithelial cells [61]. CREB1, cAMP-responsive element-binding protein 1, is a transcription factor that binds to the cAMP-response element, a DNA nucleotide sequence present in many immune-related genes, including *IL-6* [70]. LPA has been shown to induce CREB signaling in lung epithelial cells, and the conditioned medium from these cells evokes profibrotic changes in lung fibroblasts [68]. IκBα is the well-known inhibitor of NFκB transcription factor, which by default sequesters NFκB in the cytoplasm but, upon stimulation, becomes phosphorylated and allows the release of NFκB, a central mediator of the human immune response. NFκB, in the context of chronic inflammatory and autoimmune diseases, is activated by proinflammatory cytokines and drives proinflammatory cytokine, chemokine, and adhesion molecules’ production as well [71,72]. Our Luminex results indicated activation of the NFκB pathway in the presence of LPA. LPA treatment has been shown before to induce NFκB in many other circumstances [73]. In the kidney, LPA increases phosphorylation of NFκBp65, and the LPAR1 inhibitor AM095 suppresses their activation in mesangial cells [38]. Apart from ERK, it is the first time that the aforementioned signaling molecules are found to be phosphorylated upon LPA in renal TECs.

The expression of the secreted molecules identified upon LPA stimulation could be mediated through the phosphorylated signaling hub proteins we detected. All the secreted molecules that were induced by LPA (*CCL2*, *CCL3*, *CXCL8*, *CXCL10*, *ICAM1*, *IL-6*) are among the known target genes of NFκB [74] and, hence, their LPA induction could be mediated through it. Indeed, by using an NFκB-specific inhibitor, we showed that LPA induces *CCL2*, *CCL3*, *CXCL-8*, and *ICAM1* genes through NFκB. The *CXCL8* (IL-8) transcription via NFκB has been previously shown in many cell types [62,75,76,77,78,79]; in fact, the IL-8 induction from NFκB in human bronchial epithelial cells is initiated by LPA [62]. Increased expression of CCL2, at least in the context of pulmonary fibrosis, is induced by NFκB (and AP-1 subunit c-JUN) [80]. According to other studies, IL-6 is one of the highest induced NFκB-dependent cytokines in various cell types [78,81,82], however we did not observe such a regulation in the HKC-8 cell line. Concerning human primary proximal TECs, they are a potential source of IL-6, IL-8, and CCL2 in response to various proinflammatory cytokines, such as IL-1α and TNFα [72,83], which is validated by our results. Moreover, the IL-1 stimulation of IL-6, IL-8, and CCL2 in primary human PTECs and HK2 TECs is NFκB-dependent [72,84]. Additionally, NFκB induces the expression of molecules related to leukocyte recruitment/adhesion such as ICAM1 [85,86,87]. CCL2-mediated ICAM1 expression in human TECs is predominantly dependent on NFκB activation [88], while TNFα-induced activation of the *ICAM1* promoter in human endothelial cells depends on NFκB as well [89,90]. Furthermore, oxidized LDL promotes the recruitment of NFκB/p65 to the ICAM1 promoter in endothelial cells [91]. 

We detected a regulation of ICAM1 and CCL3 through JNK, which is the kinase upstream of c-JUN. ICAM1 is, indeed, regulated by c-JUN in IL-1-stimulated human primary fibroblasts [92] and TNFα-stimulated retinal-pigment epithelial cells [93]. Concerning CCL3, there are indications that it is expressed by palmitate and TNFα through JUN-involved signaling in THP-1 monocytic cells [94]. Even though it has been reported in the literature that *CCL2* and *CXCL8* are target genes of c-JUN, we did not verify this experimentally. Concerning CREB1, we found that it affects the transcription of *CCL2*, *CCL3*, *CXCL8*, and *ICAM1*. CREB1 is, indeed, required for the inducible transcription of *CXCL8* in monocytic cell lines [95]. Furthermore, it shows enriched binding to the promoter of *CCL2* in peripheral blood mononuclear cells [96]. LPA mediates CREB phosphorylation through mitogen- and stress-activated protein kinases, resulting in *CXCL8* and *CCL2* transcription in fibroblast-like synoviocytes [97]. CREB1 is involved in the expression of *CXCL8* and *CCL3* in neutrophils, too [98].

Apart from transcription factors, MAPK signaling is also implicated in the expression of the aforementioned secreted factors. Our results show that the expression of *CCL2*, *CCL3*, *CXCL8*, and *ICAM1* is MEK/ERK-dependent. Several publications are indicating the role of MEK/ERK signaling in *CXCL8* expression [99,100,101]. The MEK/ERK pathway has also been shown to mediate CCL expression. IL-13-induced CCL3 expression is dependent on ERK1/2 signaling in vivo [102]. LPS treatment augments *CCL3* transcription in vitro in bone-marrow-derived dendritic cells [103] and in vivo in the rat brain in a MEK/ERK-dependent fashion [102,104]. Moreover, TNFα or IL-1β induction of *CCL3* mRNA in rat-nucleus pulposus cells is p38- and ERK-dependent [105]. Regarding IL-6, we did not observe a MEK/ERK effect on its transcription, although several pieces of data indicate such a regulation [106,107,108]. However, corroborating our results, LPA-mediated IL-6 expression is not affected by a MEK inhibitor in microglia [109]. MEK proteins also seem to control CXCL10 expression [42,109], although we did not test this.

However, several genes encoding cytokines are simultaneously regulated by multiple signaling pathways and transcription factors. We found that *CXCL8* is regulated by MEK/ERK, NFκB, and CREB. Indeed, CREB and NFκB are among the transcription factors that are cooperatively activated for *CXCL8* transcription in human bronchial epithelial cells [77]. MEK/ERK and transcription factors AP-1 and NFκB are all involved in *CXCL8* upregulation by IL-1β in gastric-carcinoma cells [99] and by CD40 in human fetal microglia [100]. Moreover, the *P. aeruginosa*-dependent transcription of *CXCL8* in human bronchial epithelial cells is mediated by ERK signaling and a multitude of transcription factors, such as NFκB, AP-1, and CREB [77]. ICAM1 expression is controlled both by NFκB and c-JUN, upon PMA or TNFα, in endothelial cells [110]. As other studies suggest, ICAM1 expression is also regulated by both NFκB and CREB [111]. Both NFκB and ERK1/2 mediate CCL3/MIP-1a expression in the brain [104], something that we also verify in the HKC-8 cell line along with co-regulation by the JNK/JUN and CREB pathways. CCL2 and CCL3, among other chemokines, are induced by H_2_O_2_ through ERK and the nuclear translocation of NFkB, AP-1 and CREB in macrophages [112]. We also show that *CCL3* is co-regulated by MEK/ERK, JNK/JUN, NFκB, and CREB in the HKC-8 cells, whereas *CCL2* is activated by MEK/ERK, NFκB, and CREB. 

Employing divisive clustering for all the tested stimuli, LPA congregates with IL-1α, IL-1β, IL-17α, TNFα, TNFSF12/TWEAK, and CXCL14/BRAK, which are endogenous stimuli in the human body, and promethazine, PMA, FSL1, and PolyIC, which are exogenous stimuli. All the aforementioned endogenous stimuli are implicated in CKD. High levels of IL-1α are detected in renal TECs in biopsies from DN patients, while in vitro IL-1α provokes the deposition of ECM proteins [113]. IL-1β mRNA is also detected in biopsies of DN patients [114]. IL-1β contributes to systemic inflammation and the progression of modeled CKD, either type 2 diabetes-induced or adenine diet-induced, as shown by studies utilizing monoclonal anti-IL-1β in mice [114,115]. Il-1β is also produced by several cell types during IgA nephropathy and promotes inflammation and disease progression [116]. Besides, activation of the inflammasome in immune cells during kidney injury causes the secretion of IL-1α and IL-1β, which then promote cytokine and chemokine release through the IL-1 receptor (IL-1R), resulting in the further recruitment of immune cells [117]; thus, these two cytokines are important in the inflammatory component of kidney disease [118]. It is, therefore, well expected that the deletion of type I IL-1R ameliorates the early renal fibrosis induced by ureter obstruction in mice [119]. 

IL-17α levels are increased in the kidneys of diabetic mice compared to control kidneys, and treatment with anti-IL-17α antibody ameliorates renal dysfunction and disease [120]. Additionally, IL-17α positive cells have been detected in renal biopsies of hypertensive nephroangiosclerosis and kidneys of experimental hypertensive mice, while mice infused with IL-17α show higher inflammatory cell infiltration in the kidneys, with a simultaneous elevated *CCL2* and *CCL5* gene expression [121]. Moreover, it is postulated that IL-17α promotes the AKI-to-CKD transition [122]. However, studies claiming an antifibrotic role of IL-17α also exist [123,124].

TNFSF12/TWEAK is another molecule that clusters with LPA. TNFSF12/TWEAK was found to induce the secretion of CCL2, CCL3, CCL5, IL-6, IL-8, and CXCL10. Three of these molecules (CCL2, CCL5, and IL-6) have been previously shown to increase upon TNFSF12/TWEAK treatment in renal tubular cells [125], thus corroborating our results. TNFSF12/TWEAK is also implicated in renal injury [126,127,128]. Although its expression is rather low in normal kidneys, it becomes significant during tissue damage in diverse forms of AKI and CKD [129]. Increased protein expression of TNFSF12/TWEAK is detected in the renal cortex of patients with lupus nephritis (LN) and treatment of mesangial cells with TNFSF12/TWEAK promotes macrophage chemotaxis, probably through the chemotactic factors that TNFSF12/TWEAK induces [130]. Indeed, TNFSF12/TWEAK promotes the NFκB-mediated expression of proinflammatory cytokines, such as CCL2 and CCL5, in human glomerular mesangial cells [127]. Inhibiting TNFSF12/TWEAK in vivo reduces tubular chemokine expression and macrophage infiltration [125]. Blocking or deleting TNFSF12/TWEAK or its receptor induces a drop in inflammation and an improvement of renal function in several experimental models of renal disease [129,131]. On the contrary, in vivo TNFSF12/TWEAK administration leads to NFκB activation in the whole kidney and expression of chemokines from tubular cells [132]. Moreover, the development of anti-TWEAK therapies against inflammatory diseases such as RA is in progress [129]. 

TNFα is also implicated in renal function, as it directly affects the hemodynamic and excretory function of the kidney [133]. It is a potent proinflammatory cytokine, which, however, also has an immunosuppressive effect. Ιn healthy kidneys, the levels of TNFα are very low, whereas they increase in many kidney diseases upon leukocyte infiltration, as activated monocytes and macrophages are its primary source [134]. In terms of its expression, TNFα is not only expressed by the infiltrating macrophages but also by resident kidney cells such as mesangial, podocytes, and TECs [134]. TNFα can regulate proliferation and apoptosis in renal cells, but it can elicit a local proinflammatory cytokine cascade, too. In UUO, renal TNF production is increased after ureter obstruction and is implicated in tubular-cell apoptosis and interstitial fibrosis [134]. Additionally, TNFα is increased in patients with acute allograft rejection and chronic allograft nephropathy [135]. Intriguingly, in RA, TNFα has been found to induce ATX expression from synovial fibroblasts (SFs), while it induces SF activation and effector functions in synergy with LPA [136], thus proposing a possible synergism of TNFα with LPA in CKD, too.

CXCL14/BRAK promotes chemotaxis of immature dendritic cells, neutrophils, monocytes, activated human NK cells, and others [137]. CXCL14 has been detected in kidney specimens, however, it has not been extensively investigated, except for a study where CXCL14 overexpression mitigates sepsis-induced AKI, probably through the regulation of the M1/M2 macrophage ratio and the downregulation of cytokine production [138].

As most stimuli in the LPA-including cluster 2 promote CKD, we assume that LPA has a negative impact on CKD as well. Indeed, the role of LPA in CKD has been established by several studies on the mouse models of various renal pathologies. LPA is significantly increased in the urine of mice subjected to the CKD model of nephrectomy [139]. Upon UUO, LPA production from kidney explants is enhanced and LPAR1 is found to be upregulated (although LPAR2 and LPAR6 are, by default, the highest expressed LPA receptors in the kidney) [20,21]. Importantly, genetic deletion or pharmacological suppression of LPAR1 reduces tubulointerstitial fibrotic and inflammatory markers in mice subjected to UUO [20,21,140]. LPAR1 ablation decreases the number of proliferating fibroblasts and accumulating myofibroblasts induced by UUO [21]. In vitro, LPA is shown to induce CTGF expression in mouse primary proximal TECs through LPAR1 and LPAR2; CTGF then stimulates fibroblast proliferation and their differentiation to myofibroblasts, thus promoting epithelial–fibroblast communication [21].

DN is another manifestation of CKD, where LPA and the ATX/LPA/LPAR axis are implicated. LPA and LPC are significantly increased in the renal glomeruli of eNOS(^−/−^) db/db mice, a robust model of DN [141]. In the same model, LPAR1, LPAR3, and ATX-expression levels are upregulated upon disease; administration of the LPAR1/LPAR3 antagonist BMS002 ameliorates glomerular filtration and renal fibrosis, while it reduces macrophage infiltration and podocyte loss [18]. In a similar db/db model of T2D, both ATX and LPAR1 are overexpressed in the kidney cortex compared to control mice [142]. Simultaneously, another inhibitor of LPAR1/LPAR3 ameliorates albuminuria and glomerulosclerosis, the main pathological feature of type 2 DN [142]. Similarly, an LPAR1 inhibitor, AM095, inhibits the expression of proinflammatory cytokines and fibrotic factors in the kidney, reduces glomerular matrix expansion, and improves kidney function in a streptozotocin-induced type 1 diabetic model [38]. In a mesangial cell line, LPA significantly increases the expression of proinflammatory cytokines TNFα, IL-1β, IL-6, and CCL2/MCP-1 and promotes phosphorylation of NFκB and JNK [38], while at the same time it induces the profibrotic factors TGFβ1 and fibronectin in a glycogen synthase kinase (GSK)3B and sterol regulatory element-binding protein (SREBP1)-mediated fashion [142]. In our study, we have gone one step further, showing that LPA promotes the expression of proinflammatory molecules in TECs as well.

Beyond cluster 2, the stimulus with the most responders was HGF, which belongs to cluster 1. Intriguingly, HGF seems to suppress chronic renal failure, and administration of HGF improves renal fibrosis [143]. Another molecule of interest is one of the most common secreted factors identified in our experiments, TNFSF10/TRAIL. Experimental and clinical studies have illustrated that TNFSF10/TRAIL is up-regulated in different kidney diseases, both in DN and in non-diabetic conditions such as LN, rejected kidney transplant, AKI, and others [129]. The TNFSF10/TRAIL receptor, TRAIL-R2, has been pointed out as the protein most strongly associated with the decline of kidney function [129].

Finally, apart from the previously mentioned instances, a number of our results can be verified by the existing data in the literature. According to a study, the addition of LPA in HKC-8 cells induces ERK1/2 phosphorylation in these cells [144], corroborating our results where LPA 18:1 induced phosphorylation of MEK, of the kinase upstream of ERK, and of ERK, albeit not statistically significantly. Other studies also support our results, as HKC-8 cells have been found to respond to BMP-7 by reversing TGFβ1-induced EMT [145,146]. In our assay, HKC-8 cells also respond to BMP-7 by inducing phosphorylation of GSK3B. In an in vitro model studying kidney fibrosis, it has been found that upon injury with cisplatin, HΚC-8 cells secrete CCL5 and IL-6 [147]. Our Luminex data indeed show that HKC-8 cells are able to secrete these cytokines, as both CCL5 and IL-6 were induced upon a series of stimuli. In another setup, EGF activated EGFR, p38 MAPK, NFκBp65, and STAT3, leading to inducible nitric oxide synthase expression in HKC-8 cells [148]. In our experiment, human EGF drove many protein phosphorylations, including EGFR, JUN, MEK1, ERK1, and others. However, it did not activate STAT3 or MK12 (p38γ).

Conclusively, in this report, we have identified the responses of human renal proximal TECs to a series of 176 immunological stimuli. The subsequent quantification of the levels of 27 intracellular phosphoproteins and 32 extracellular molecules with multiplex ELISA reveals a large amount of information concerning the signaling and physiology of renal proximal tubular epithelial cells and their possible interaction with resident stromal cells. Among the 176 stimuli, LPA stands out as a proinflammatory stimulus promoting the phosphorylation of important signaling hubs and the secretion of factors of clinical relevance concerning CKD. Finally, our results offer some mechanistic insight into the contribution of LPA to kidney-related chronic inflammation and further pinpoint the ATX/LPA axis as crucial in the development of renal pathology and as a possible therapeutic target. 

### Limitations of the Study

Among the limitations of the study is the fact that the main multiple ELISA experiment with the 176 stimuli was performed in single wells, instead of triplicates, however, this was a high-throughput experiment that would be difficult to have been performed in triplicates. Moreover, the data of the LPA response and the response on some other stimuli have been verified with further experiments done in triplicate wells. Another limitation is the fact that the inhibitors used against LPARs or signaling molecules may have been added in excess, despite our efforts to adhere to concentrations used in several other publications. Finally, we realize that our experiments were done in vitro, and, therefore, in an artificial environment deprived of the extracellular matrix and neighboring cells, which poses another limitation.

## 4. Materials and Methods

### 4.1. Cell Culture and Cell Stimulation with 176 Immune Molecules

HKC-8 cells, provided by Roche, were grown in DMEM:F12, glutamine 2 mM, insulin-transferrin-selenium (ITS) supplement 1×, FBS 2.5%, penicillin 100 u/mL, streptomycin 100 μg/mL, and amphotericin B 2.5 μg/mL. They were seeded at a cell density of 24,000 cells/well in 96-well plates and left to attach overnight. Starvation followed next day with DMEM:F12, glutamine 2 mM, ITS supplement 1×, BSA 0.2%, penicillin 100 u/mL, and streptomycin 100 μg/mL for 3 h. The medium was replaced (again with starvation medium) and the addition of multiple stimuli followed, as shown in the Supplementary Materials. Two experiments took place. The first experiment was designed to measure the intracellular phosphorylation events; hence, we used cell lysates. The second experiment was designed to measure secreted factors such as cytokines; therefore, we used cell supernatants. The phosphorylation events were tested at two time points, meaning that this experiment involved two sub-experiments. 

The stimuli were added at the same concentrations between the two experiments but at different volumes. In total, 5 μL of diluted stimuli were added in 20 μL of medium in each well of the phosphoprotein plates, and 20 μL of stimuli were added in 80 μL of medium in each well of the secreted factors’ plates. In total, 175 stimuli were added apart from LPA and the controls. For each experiment (phosphoproteins/secreted factors), the 175 stimuli and LPA 18:1 were added in single wells, while medium (as control) was added in 5 wells separated in the 2 plates. For the repetition of LPA stimulation, three LPA species were used, each of them added in triplicate wells and chloroform (LPA’s solvent) again in triplicate wells. Before the addition, LPA and chloroform were heated so as to be easily diluted in the cell medium.

Between the phosphoprotein and secreted-factors experiments, the incubation time with the stimuli differed: 5 min for one set of plates for the phosphoprotein experiment, 25 min for another set of plates for the phosphoprotein experiment, and 24 h for the secreted factors experiment. The different incubation times reflect the different times necessary for phosphorylation events and expression (transcription and translation) to take place.

### 4.2. Multiplex ELISA

#### 4.2.1. Phosphoprotein Experiment

After the 5 or 25-min incubation period, each plate was placed on an ice pack in order to stop the reactions in all wells simultaneously, and cells were lysed by adding 40 μL of lysis buffer mix in each well. The lysis buffer mix contained a ProtATonce custom-made lysis buffer and phosphatase inhibitors, a protease inhibitors cocktail, and extra phenylmethylsulfonyl fluoride (PMSF). Lysis took place by shaking the plate at 4 °C for 20 min while keeping it continuously on the ice pack. The plates with the lysates were stored at −20 °C, covered with aluminum plate sealers. Prior to the Luminex assay, the lysates were thawed and sonicated (4 times, 10 s each) and the plates were spun down at 2700 g for 20 min. The top 50 μL of the samples were transferred into flat-bottom 96-well plates containing 50 μL of bead mix per well, pre-washed with Assay Buffer (PBS with 1% BSA, pH 7.4). The bead mix contained magnetic beads internally dyed with precise proportions of red and infrared fluorophores. The differing proportions of the red and infrared fluorophores result in unique spectral signature microspheres. Each unique microsphere-bead was conjugated with a distinct mAb against a phosphoprotein, thus allowing simultaneous recognition of 27 phosphoproteins in one sample.

Each plate, covered with a sealer, was shaken at maximum speed for 90 min and then placed on a magnetic separator that keeps the magnetic beads down and allows discarding of the supernatant. The bead–sample mix was washed twice with the assay buffer in this manner and the biotinylated second/detection antibody was added in all wells. Incubation with the second antibody lasted 90 min, with the sealed plates shaking at maximum speed. Extra assay buffer was added and the beads were washed twice in order to remove the excess antibody. A Streptavidin and R-Phycoerythrin Conjugate mix diluted in assay buffer was used at 5 μg/mL, and a volume of 50 μL was added per well. Following a 15 min shaking incubation, the supernatant was discarded, the beads were washed with assay buffer, and, finally, 130 μL of assay buffer was added per well prior to the measurement.

#### 4.2.2. Secreted-Factors Experiment

After the 24-h incubation, the plates were spun down (200 g for 5 min) and the supernatants were transferred into new plates, covered with an aluminum plate sealer and stored at −20 °C. On the day of the Luminex assay, 50 μL of the samples were transferred into flat-bottom 96-well plates, containing the bead mix conjugated with mAbs against 32 secreted factors. The same process followed as described for the phosphoproteins.

All measurements were taken in a Flexmap 3D of Luminex corporation. Significant effort was devoted to maximizing the number of measurements that could be obtained from each sample of cells: a 96-well plate assayed for 27 phosphoproteins yielded 2592 measurements, and a plate assayed for 32 secreted factors yielded 3072 measurements.

### 4.3. Bioinformatic Analysis

#### 4.3.1. Luminex-Data Preprocessing

Independent analysis was done for the phosphoprotein experiment and the secreted factors experiment. In both experiments, the control wells, treated with plain medium, were distributed between the different plates and the median of the multiple control wells was calculated. The response to the plain medium (control wells) was considered as the unstimulated state, whereas the response to a stimulus was considered as the stimulated state. For each phosphoprotein or secreted factor induced by a certain stimulus, the measurement at the stimulated state (usually a single measurement or the median value in case of replicates, e.g., the triplicate LPA wells for each LPA species) was divided with the measurement at the unstimulated state (median of the control wells). The emerging ratio corresponds to the fold-change in the response of each measured component (phosphoprotein/ secreted factor) to the specific stimulus, compared to the response to plain medium. The distribution of the fold changes seen in the responses is depicted in Supplementary Figure S1A. 

In order to call a signal (the ratio of stimulated to unstimulated state) active or not, we used a threshold of fold change at 1.5. The choice of threshold was made through a sensitivity analysis on the effect of the threshold on the signals dataset. In particular, the percentage of activations was recorded at several thresholds. At low thresholds, slight changes in the threshold greatly affected the number of activations, whereas, at higher thresholds, the dataset was rather insensitive to threshold changes. Therefore, we decided to set the threshold at 1.5, where the number of activations is rather stable, but there are also enough activations for the subsequent analysis (Supplementary Figure S1B).

All the responses were processed using the open-access MATLAB-based software DataRail (http://code.google.com/p/sbpipeline/wiki/DataRail (version v1.3, accessed on 1 June 2014)). 

#### 4.3.2. Clustering

Phosphoprotein and secreted factors fold changes were combined and stimuli not causing any signal activation were removed, along with globally non-responsive signals. Gower’s metric was used to define pairwise-stimuli distances post fold change to binary values’ transformation (1: activated; 0: non-activated). Divisive clustering was performed based on the calculated distance matrix. Gower’s distance was calculated using the proxy R package, while divisive clustering was performed using cluster R package functions.

#### 4.3.3. Pathway Analysis

Cluster 2 stimuli causing at least one activation event and signals responding at least once were concatenated. Elements not being coded by a gene were filtered out, with the exception of LPA 18:1, which was replaced by *ENPP2*. ClusterProfiler R package [149] was used for over-representation analysis of both GO terms and KEGG pathways using default parameters. An FDR-corrected *p*-value threshold of at least 0.05 was applied to define significantly enriched terms.

#### 4.3.4. Microarray-Data Re-Analysis

Raw microarray data were fetched from GEO series GSE66494 (Agilent 4x44K G4112F) and GSE104066 (Affymetrix HuGene-2_1-st) using the GEOquery R package [150]. Agilent microarrays were background corrected using the normexp method with a 50 offset, as suggested by limma package authors [151], and then quantile normalized between arrays. Affymetrix data were background corrected and RMA normalized using the oligo R package [152]. Both datasets were quality-controlled post normalization, using arrayQualityMetrics R package [153] and Principal Component Analysis (PCA). One (GSM1623315) and two (GSM3904846, GSM2788881) outlier samples were filtered from GSE66494 and GSE104066, respectively. Control probes and probes matching to either no or multiple HGNC gene symbols were not considered for downstream analysis, along with those having intensity values close to the background. Agilent probes with a high cross-linking potential were not maintained either. Probe-intensity values were summarized at the gene level, and weighted mean value was calculated in one:many gene:probe relationships. Differential-expression analysis was performed using an empirical Bayes statistic as implemented in limma package. Absolute fold change ≥ 1.2 and FDR corrected *p*-value < 0.05 were set as differential expression thresholds.

#### 4.3.5. Single-Cell Data Analysis

Single-cell data were downloaded from zenodo (https://doi.org/10.5281/zenodo.4059315 (accessed on 15 November 2021)). Pre-processed CD10^+^ and CD10^−^ objects were log-normalized and integrated using Seurat package v4.0.5 using a pre-computed AnchorSet based on 2000 features [154]. Biological annotation of the original publication was maintained. Marker genes and differential expression analysis was performed using FindMarkers function under default parameters on the RNA assay of the integrated object. Absolute fold change ≥ 1.2 and adjusted *p*-value < 0.05 were set as significance thresholds.

Cell-to-cell analysis was performed by SingelCellSignalR package v1.4.0 [155]. Cells were grouped per cellular population, except for proximal tubule positive (PT-pos; CD10^+^) and proximal tubule negative (PT-neg; CD10^−^) cells. SingleCellSignalR default-ligand-receptor database was extended to include some interactions of interest, as described in CellTalkDB v0.0.1 [156]. Circos plots were visualized using circlize package v0.4.13 [157].

### 4.4. RNA Analysis

#### 4.4.1. LPA Stimulation—RNA Isolation—Reverse Transcription—RT-qPCR

HKC-8 cells were seeded in 6-well plates at a density equal to 300,000 cells/well with a subsequent overnight starvation (medium with 0.2% BSA and no FBS). Cells were stimulated with 2.5, 5, or 10 μM LPA 18:1 (Avanti Lipids, Sigma-Aldrich, Merck, St Louis, MO, USA), dissolved in starvation medium. As a control, plain starvation medium or starvation medium with the LPA’s solvent, chloroform, was used. Cells were incubated at 37 °C and 5% CO_2_ for 1, 4, 12, or 24 h prior to RNA extraction. RNA extraction was done with TRI Reagent (TR118, MRC, Cincinnati, OH, USA) in accordance with the instructions of the manufacturer, with a slight modification at the RNA-precipitation stage, where glycogen was added. The RNA concentration and purity were determined with NanoDrop^®^ ND-1000 UV-Vis Spectrophotometer (Thermo Fisher Scientific, Waltham, MA, USA), calculating the optical density ratio at wavelengths of 260/280 nm and 260/230 nm. Samples were placed at −80 °C until further use. First-strand cDNA was generated with the Moloney murine leukemia virus reverse transcriptase (28025-013, Invitrogen, Thermo Fisher Scientific, Waltham, MA, USA) using 2 μg of RNA and according to the reagent’s protocol. Real-time PCR was performed on a BioRad CFX96 Touch™ Real-Time PCR Detection System (Bio-Rad, Hercules, CA, USA), using SYBR Select Master Mix (4472913, Thermo Fisher Scientific, Waltham, MA, USA), 25 ng of each cDNA per reaction, and primers that are listed in Table S3. The thermal-cycling conditions for 40-cycles amplification were at 95 °C for 10 s and 58, 59, or 60 °C for 45 s. Normalization of the Ct values was done against the reference gene *B2M*. The relative quantification of the target-gene expression was done using the Livak (2^−ΔΔCq^) method and presented as fold change of each normalized target gene in the LPA-treated samples relative to control samples. The statistical analysis between groups of the dose-response study was performed using Brown–Forsythe’s and Welch’s ANOVA tests or the Kruskal–Wallis test in case of non-normal distribution. Two-way-ANOVA was performed for the time-course experiments. Finally, in the case of LPARs expression, the 2^−ΔCq^ formula was used in order to compare the levels of the different *LPARs*. Statistical analysis was done with GraphPad.

#### 4.4.2. Experiments with LPAR or Phosphoprotein Inhibitors

HKC-8 cells were seeded and starved as above and pretreated with inhibitors for one hour. LPAR1/3 inhibitor (Ki16425, Cat. no: HY-13285, MedChemExpress, Monmouth Junction, NJ, USA) was added at 10 μM; LPAR2 inhibitor (H2L5186303, Cat. no: 10-1452, Focus Biomolecules, Plymouth Meeting, PA, USA) was added at 10 μM; CREB inhibitor (666-15, Cat. no: A616443, Toronto Research Chemicals, North York, ON, Canada) was added at 10 μM; JNK inhibitor (SP600125, Cat. no: 420119, Calbiochem, San Diego, CA, USA) was added at 50 μM; NFκB inhibitor (JSH-23, Cat. no: HY-13982, MedChemExpress, Monmouth Junction, NJ, USA) was added at 100 μM; and MEK/ERK inhibitor (PD98059, Cat. no: 513000, Calbiochem, San Diego, CA, USA) was added at 50 μM. As controls, some wells were treated with plain medium or with an equivalent volume of DMSO, the solvent of the inhibitors. After 1 h, and without removing the inhibitor or DMSO, LPA was added to the experimental wells to a final concentration of 10 μM for 4 h. RNA isolation, reverse transcription, and RT-qPCR were performed as above. The statistical analysis was done with unpaired *t*-test or Welch’s t-test, depending on the equality of standard deviation between the different groups, or Mann–Whitney in the case of non-normal data. Statistical analysis was done with GraphPad.

### 4.5. Image Creation

Images of Figure 1 and Figure 9 were created with BioRender.com, with agreement numbers OH23ZPFMT5 and CX23ZPEU47, respectively. BioRender.com was accessed on 1 June 2022.

## Data Availability

The data presented in this study are available on request from the corresponding author.

## Supplementary Materials

The following supporting information can be downloaded at: https://www.mdpi.com/article/10.3390/ijms23137452/s1.

## Abbreviations

ANG1: angiopoietin; ARPCs, adult renal progenitor cells; ATX, autotaxin; BDNF, brain-derived neurotrophic factor; BMP2, bone morphogenetic protein 2; CKD, chronic kidney disease; CNTF, Ciliary Neurotrophic Factor; CREB1, cAMP responsive element binding protein 1; CTGF, connective tissue growth factor; DN, diabetic nephropathy; ECM, extracellular matrix; EMT, epithelial to mesenchymal transition; FC, fold change; GFR, glomerular-filtration rate; GN, glomerulonephritis; GSK, glycogen synthase kinase; HGF, hepatocyte growth factor; ICAM1, Intercellular Adhesion Molecule 1; IL, interleukin; IP-10, 10-kDa interferon-inducible protein; ITS, insulin-transferrin-selenium; JNK, c-Jun N-terminal kinase; LFA1, lymphocyte-function-associated antigen-1; LN, lupus nephritis; LPA, lysophosphatidic acid; LPAR, LPA receptor; MCP1, monocyte chemoattractant protein-1; MIP-1α, macrophage inflammatory protein-1α; NK, natural killer; PDGFB, platelet-derived growth factor; PMA, phorbol 12-myristate 13-acetate; PROK1, Prokineticin 1; PT, proximal tubule; RA, rheumatoid arthritis; RANTES, regulated on activation, normal T cell expressed and secreted; SASP, senescence-associated secretory phenotype proteins; SFs, synovial fibroblasts; SREBP1, sterol regulatory element-binding protein; T2D, type 2 diabetes; TECs, tubular epithelial cells; TGFβ, transforming growth factor beta; TLR, toll-like receptor; TNFα, tumor necrosis factor alpha; TSLP, thymic stromal lymphopoietin; UUO, unilateral ureter obstruction; WISP1, WNT1-inducible-signaling pathway protein 1.
