# Supplementary material for: Lysophosphatidic Acid Is a Proinflammatory Stimulus of Renal Tubular Epithelial Cells
Source: Int J Mol Sci. 2022 Jul 5;23(13):7452. doi: 10.3390/ijms23137452 (PMC9267536; doi:10.3390/ijms23137452)
Supplement: Supplementary file 1 [file ijms-23-07452-s001.zip › ijms-1789504-supplementary.pdf]

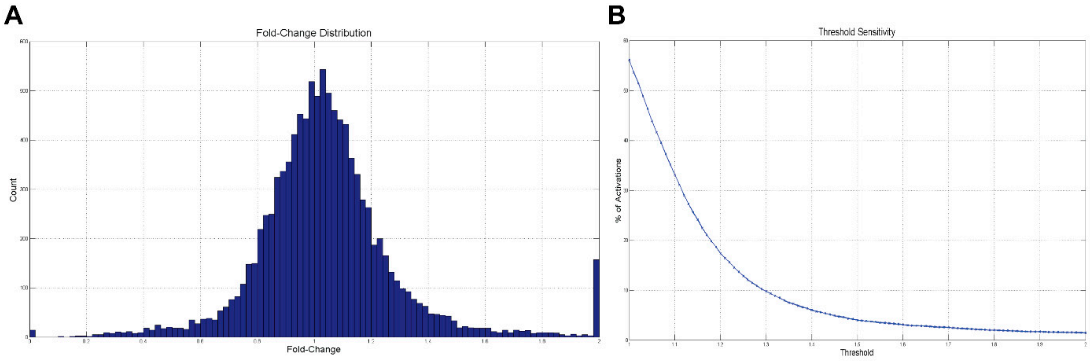

Figure S1. Active signal threshold selection. Related to Figures 2 and 3. **A.** Distribution of the fold changes (ratio of the fluorescence at the stimulated versus the unstimulated state) for all signals. The median of responses upon stimulation with medium was considered as the unstimulated state fluorescence value. **B.** Sensitivity analysis on the effect of the fold change threshold on the dataset. At low thresholds, slight changes in the threshold greatly affect the number of activations (percentage of fold change values exceeding the threshold), whereas at higher thresholds the dataset is rather insensitive to changes of the threshold. Activation threshold was selected as the fold change value in which the dataset was adequately insensitive accompanied by enough activations for downstream analysis.

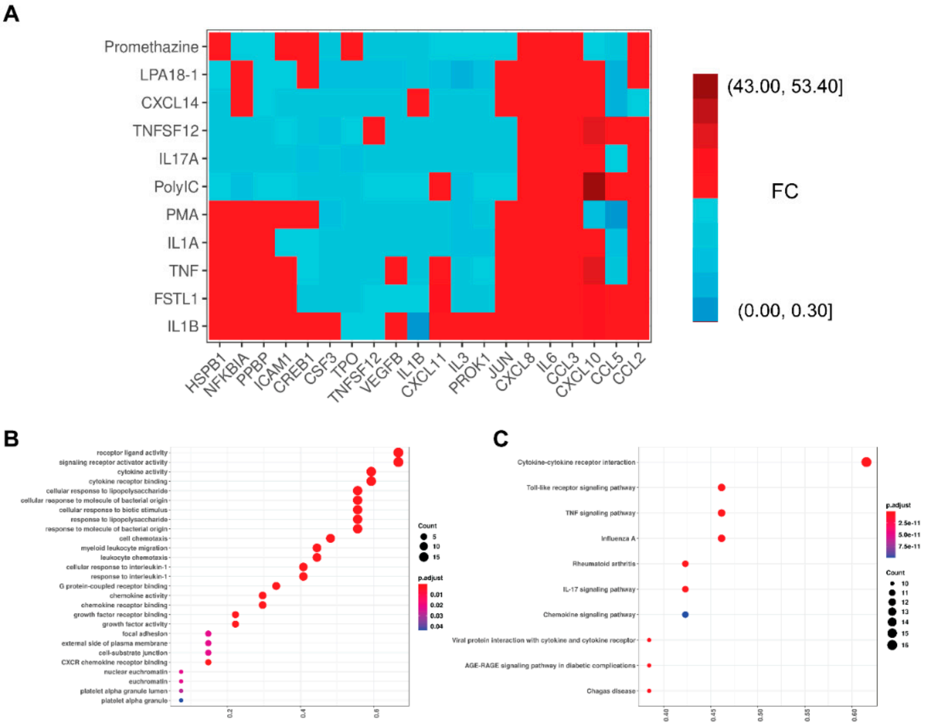

Figure S2. LPA clusters with proinflammatory stimuli. Related to Figure 6. **A.** Cluster 2 heatmap. Signals with no response in the cluster were filtered out. **B, C.** Over-representation analysis of cluster 2 signals and responses using GO and KEGG as background database, respecttively, suggest that cluster 2 comprises of proinflammatory molecules. Signals and responses with no intra-cluster effect/response were not considered during pathway analyses. Top 10 enriched terms of each GO semantic category (Molecular Function, Biological Process and Cellular Component) and KEGG database are shown.

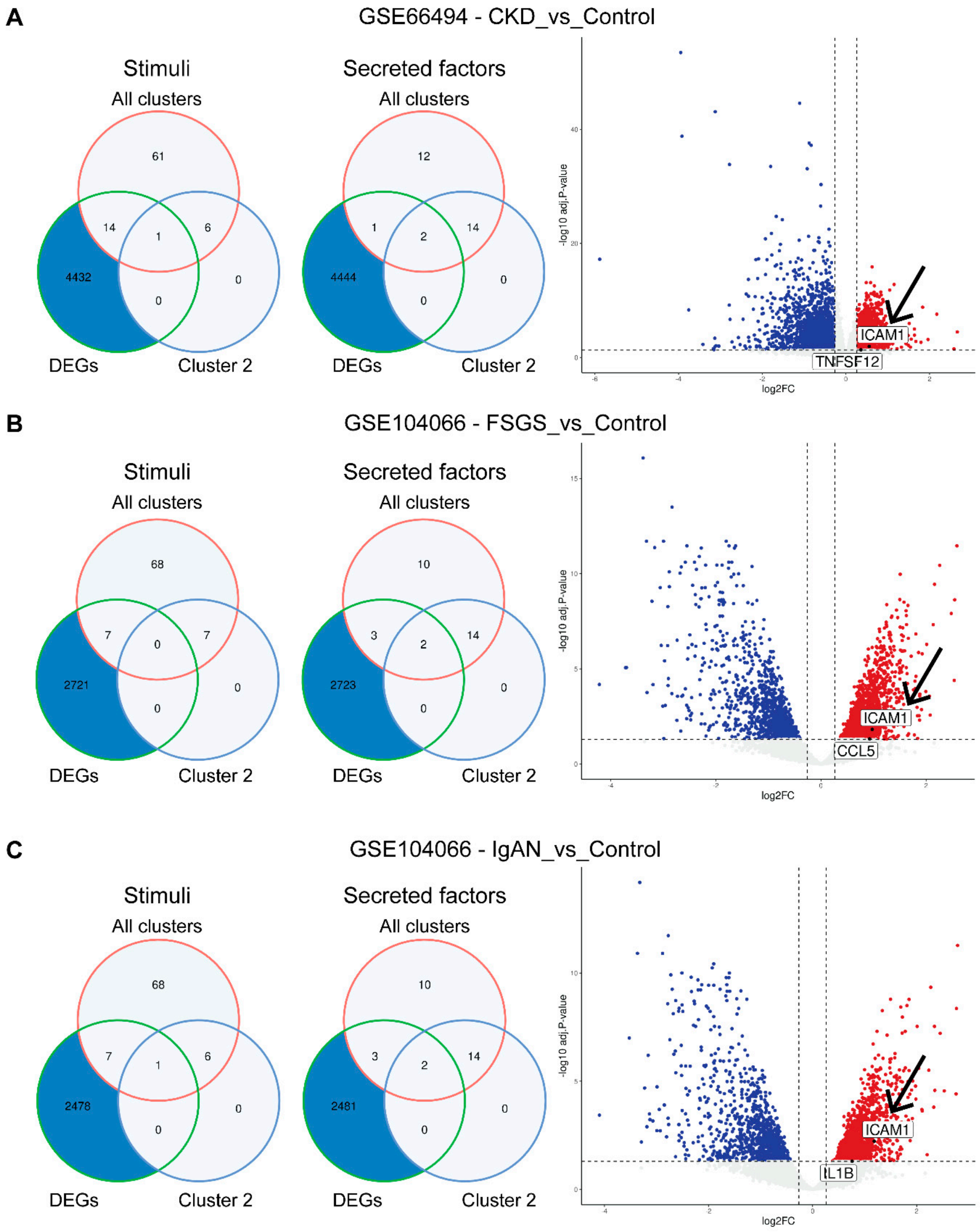

Figure S3. Expression of cluster 2 targets upon kidney disease. Related to Figure 6. Reanalysis of two microarray datasets including three kidney pathological phenotypes and control samples. Dataset GSE66494 in **A** and dataset GSE104066 in **B**. Venn diagrams depict the inter-section of differentially expressed genes (DEGs) ((│FC│≥1.2), Adjusted p-value<0.05) of each comparison with active ELISA stimuli and responders (secreted factors). Intersected DEGs are labeled at the volcano plots. Arrows indicate the molecules responding to LPA. TNFSF12 and IL1B1 are both cluster 2 stimuli and responders. *CKD, Chronic Kidney Disease; FGS, Focal Segmental GlomeruloSclerosis; IgAN, Immunoglobulin A Nephropathy.*

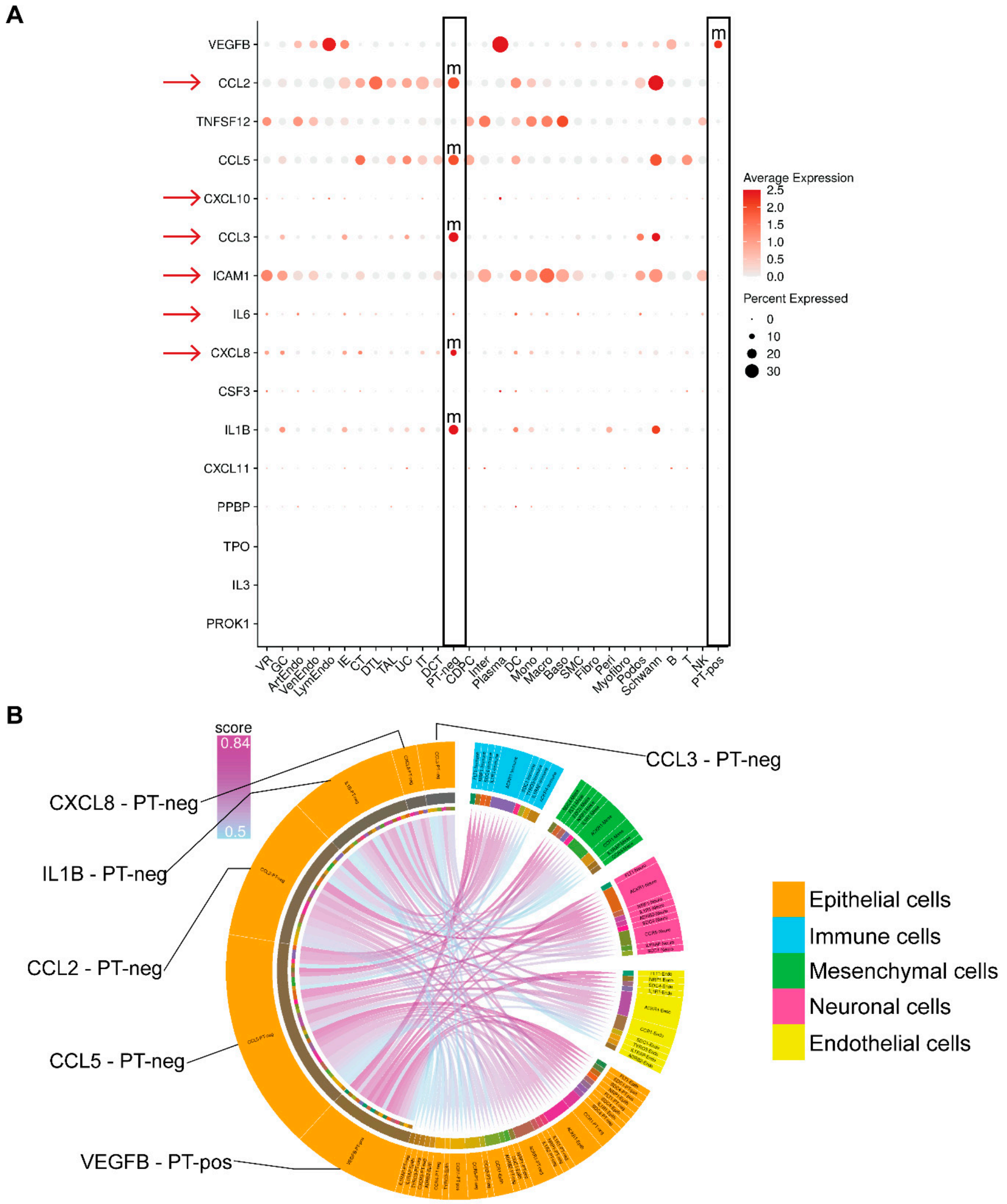

Figure S4. Cell specificity of cluster 2-induced biological factors and their cell targets. Related to Figure 6. **A. Dot Plot of cluster 2 responders.** Marker genes of PT-pos ($CD10^+$) and PT-neg ($CD10^-$) cells are annotated with the letter m. LPA responding features are pinpointed with a red arrow. TPO, IL3 and PROK1 were not detected at the single cell level. Markers are discovered using a Wilcoxon rank-sum test (FC≥ 1.2, adjusted p-value <0.05). **B. Ligand-receptor (cell-to-cell) analysis of proximal tubule marker ligands.** Interactions depicted are focused on cluster 2 responders secreted from and marking proximal tubule cells. The analysis was performed on cells of both phenotypes. Circos plot segments are named after the respective feature (ligand/receptor) and cell type/population name. *PT: Proximal Tubule cells.*

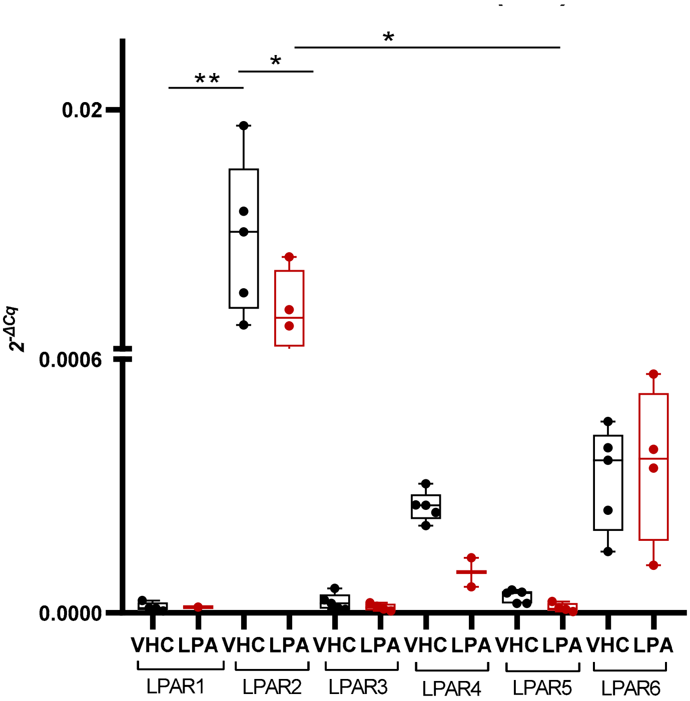

Figure S5. Endogenous gene expression levels of LPA receptors (LPARs) in HKC-8 cells. Related to Figure 7. Cells were stimulated with LPA 18:1 or the equivalent volume of chloroform (Vhc), RNA was extracted and reverse transcribed. LPAR levels were quantified with RT-qPCR, normalized with *B2M* and compared using the $2^{-\Delta Cq}$ values. Statistical analysis was done with Kruskal-Wallis test. *$p<0.05$, **$p<0.01$.

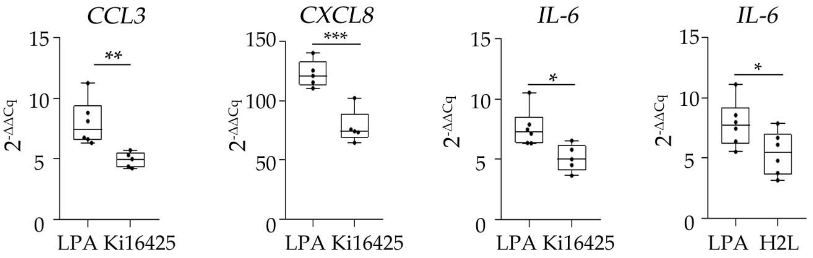

Figure S6. LPARs mediating LPA signaling. Related to Figure 8. HKC-8 cells were pretreated for 1 hour with LPAR1/3 inhibitor (Ki16425) 10μM or LPAR2 inhibitor (H2L5186303) 10μM and then treated with LPA at a final concentration of 10 μM for four hours. Statistical analysis was performed with unpaired t-test or Welch's test in the case of normal data and with Mann-Whitney in the case of non-normal data. *p<0.05, **p<0.01, ***p<0.001.

Table S1. Related to Figures 2 & 3. Stimuli of HKC-8 cells. Supplier, catalogue number and target concentration are provided.

Supplier	Stimulus	Cat Number	Target C (ng/ml)
InvivoGen	Pam3CSK4	tlrl-kit1hw	1000
InvivoGen	Poly(I:C) HMW	tlrl-kit1hw	10000
InvivoGen	LPS-EK standard	tlrl-kit1hw	10000
InvivoGen	FLA-ST standard	tlrl-kit1hw	1000
InvivoGen	FSL1	tlrl-kit1hw	1000
InvivoGen	Imiquimod	tlrl-kit1hw	1000
InvivoGen	ssRNA40/LyoVec	tlrl-kit1hw	1000
InvivoGen	C12-iE-DAP	tlrl-nodkit	1000
InvivoGen	MDP	tlrl-nodkit	10000
InvivoGen	Tri-DAP	tlrl-nodkit	10000
PeproTech	IL17A	200-17	100
PeproTech	NT-3	450-03	200
PeproTech	NT-4	450-04	100
PeproTech	SCF	300-07	100
PeproTech	SCGF-A	100-22A	500
PeproTech	SFAS ligand	310-03H	10
PeproTech	Soluble RANK ligand	310-01	100
PeproTech	TGFB1	100-21	10
PeproTech	TGFB2	100-35B	10
PeproTech	TGFB3	100-36E	10
PeproTech	VCAM-1	150-04	100
PeproTech	VEGF 121	100-20A	100
PeproTech	WISP-3	120-20	150
PeproTech	ANG-1	130-06	250
PeproTech	ANG-2	130-07	500
PeproTech	GMF-beta	450-37	100
PeproTech	CCL2	300-04	200
PeproTech	CCL3	300-08	200
PeproTech	CCL4	300-09	1000
PeproTech	CCL5	300-06	500
PeproTech	SDF-1A	300-28A	200
PeproTech	SDF-1B	300-28B	200
PeproTech	GM-CSF	300-03	20
PeproTech	IFN-G	300-02	100
PeproTech	IL-1A	200-01A	50
PeproTech	IL-1B	200-01B	50
PeproTech	IL-10	200-10	10
PeproTech	Human EGF (Animal Free)	AF-100-15	100

PeproTech	Beta-NGF	450-01	50
PeproTech	Activin A	120-14	100
PeproTech	DKK-1	120-30	100
PeproTech	IFN beta	300-02BC	50
PeproTech	IL-12	200-12	50
PeproTech	IL17F	200-25	100
PeproTech	IL22	200-22	200
PeproTech	IL23	200-23	100
PeproTech	PDGF-AA	100-13A	50
PeproTech	PDGF-AB	100-00AB	100
PeproTech	PDGF-BB	100-14B	50
PeproTech	IL16 (121)	200-16A	100
PeproTech	IL6	200-06	100
PeproTech	TNFA	300-01A	100
PeproTech	IL-13	200-13	100
PeproTech	CXCL14/BRAK	300-50	200
PeproTech	CCL4L1/LAG1	300-58	200
PeproTech	CCL3L1/LD78B	300-56	200
PeproTech	CCL28/MEC	300-57	50
PeproTech	CCL19/MIP3B	300-29B	500
PeproTech	Amphiregulin (98)	100-55B	100
PeproTech	BMP-13/CDMP-2	120-04	300
PeproTech	BMP-3	120-24B	100
PeproTech	CTGF	120-19	50
PeproTech	CTGFL/WISP-2	120-16	50
PeproTech	CYR61	120-25	200
PeproTech	EG-VEGF	100-44	100
PeproTech	Endostatin	150-01	1000
PeproTech	Epigen	100-51	400
PeproTech	FGF-10	100-26	500
PeproTech	FGF-16	100-29	100
PeproTech	FGF-17	100-27	100
PeproTech	FGF-18	100-28	100
PeproTech	FGF-21	100-42	10
PeproTech	FGF-23	100-52	50
PeproTech	FGF-4	100-31	100
PeproTech	FGF-5	100-34	50
PeproTech	FGF-6	100-30	250
PeproTech	FGF-8	100-25	100
PeproTech	FGF-9	100-23	100
PeproTech	FGF-acidic	100-17A	100

PeproTech	Galectin-1	450-39	100
PeproTech	Galectin-3	450-38	20
PeproTech	GDF-11	120-11	10
PeproTech	IL4	200-04	100
PeproTech	HGF	100-39	50
PeproTech	BDNF	450-02	50
PeproTech	Klotho	100-53	50
PeproTech	NOGGIN	120-10C	200
PeproTech	OPG	450-14	10
PeproTech	PDGF-CC	100-00CC	125
PeproTech	PEDF	130-13	200
PeproTech	sCD100	310-29	50
PeproTech	sCD14	110-01	130
PeproTech	sCD40 ligand	310-02	500
PeproTech	SDLL-1	140-08	500
PeproTech	SDLL-4	140-07	500
PeproTech	SFRP-1	120-29	100
PeproTech	Sonic HedgeJoq	100-45	200
PeproTech	SPARC	120-36	100
PeproTech	TGFA	100-16A	200
PeproTech	CNTF	450-13	200
PeproTech	GDNF	450-10	200
PeproTech	TRAIL	300-59	100
PeproTech	TWEAK	310-06	100
PeproTech	VEGF 165	100-20	100
PeproTech	VEGF-B	100-20B	100
PeproTech	Visfatin	130-09	250
PeproTech	WISP-1	120-18	200
PeproTech	WNT-7A	120-31	25
PeproTech	HB-EGF	100-47	50
PeproTech	IGF-BP2	350-06B	500
PeproTech	IGF-BP4	350-05B	100
PeproTech	IGF-BP5	100-05	100
PeproTech	IGF-BP7	350-09	30
PeproTech	IGF-II	100-12	100
PeproTech	CDNF	450-05	10
PeproTech	MANF	450-06	100
PeproTech	Midkine (MK)	450-16	100
PeproTech	Pleiotrophin	450-15	100
PeproTech	Slit2-N	150-11	500
PeproTech	APRIL	310-10C	200

PeproTech	BMP-2	120-02	100
PeproTech	BMP-4	120-05ET	200
PeproTech	BMP-6	120-06	300
PeproTech	BMP-7	120-03	100
PeproTech	FGF-20	100-41	20
PeproTech	G-CSF	300-23	100
PeproTech	GDF-7	120-37	100
PeproTech	IL-11	200-11	100
PeproTech	WNT-1	120-17	50
PeproTech	Betacellulin	100-50	50
PeproTech	IL-15	200-15	100
PeproTech	IL19	200-19	100
PeproTech	IL20	200-20	200
PeproTech	IL9	200-09	30
PeproTech	KGF	100-19	100
PeproTech	IL17B	200-28	100
PeproTech	IL17D	200-27	200
PeproTech	IL17E	200-24	100
Prestwick	Dexamethasone	Prestw-0130	392
Prestwick	Riluzole hydrochloride	Prestw-0167	4006
Prestwick	Norethindrone	Prestw-0253	3999
Prestwick	Clomipramine hydrochloride	Prestw-0269	4005
Prestwick	Clenbuterol hydrochloride	Prestw-0345	4015
Prestwick	Betaxolol hydrochloride	Prestw-0382	3989
Prestwick	Bisacodyl	Prestw-0419	3975
Prestwick	Cisapride	Prestw-0430	4007
Prestwick	Digitoxigenin	Prestw-0436	3970
Prestwick	Progesterone	Prestw-0477	4025
Prestwick	Serotonin hydrochloride	Prestw-0481	5901
Prestwick	Benperidol	Prestw-0484	3967
Prestwick	Betahistine mesylate	Prestw-0543	5648
Prestwick	Hexestrol	Prestw-0699	4001
Prestwick	Tolmetin sodium salt dihydrate	Prestw-0856	3519
Prestwick	Carbachol	Prestw-0880	3982
Prestwick	Promethazine hydrochloride	Prestw-0888	3979
Prestwick	Nisoxetine hydrochloride	Prestw-0910	3528
Prestwick	Menadione	Prestw-NAT-0101	3995
Prestwick	Irinotecan hydrochloride trihydrate	Prestw-1494	58668
Prestwick	Hydrocotarnine	Prestw-NAT-0153	3989
Prestwick	Procyclidine hydrochloride	Prestw-0844	3564
Prestwick	Digoxin	Prestw-0437	4061

Prestwick	Metacycline	Prestw-0964	3716
Prestwick	Beta-Escin	Prestw-0676	3620
SantaCruz	Epigallocatechin-3-gallate (EGCG)	sc-200802	45840
SantaCruz	Mepyramine	sc-203629	2854
SantaCruz	Lovastatin	sc-200850	3959
SantaCruz	High glucose	sc-211203	9008000
SantaCruz	Forskolin / Coleonol	sc-3562	4105
SantaCruz	Staurosporine	sc-3510	466
Sigma	PMA (Phorbol 12-myristate 13-acetate)	P1585	500
Sigma	Insulin	I9278	1000
Sigma	Formaldehyde	15512	150
Sigma	NaCl	S5886	8766000
Sigma	H_2O_2	H3410	17005
VWR	Dimethyloxalylglycine	CAYM71210-10	175140

Table S2. Related to Figures 2-5. Lipid stimuli. Supplier, catalogue numbers and target concentrations are provided.

Supplier	Stimulus	Cat Number	Target C (μM)
BAvanti	LPA 18:1	857130	10
Avanti	LPA 16:0	857123	10
Avanti	LPA 20:4	857125	10

Table S3. Related to *RNA analysis*. Primers for qPCR of human genes.

Target Gene	Forward Primer (5′→3′)	Reverse Primer (5′→3′)
B2M	CTGAAAAAGATGAGTATGCC	ACCCTACATTTTGTGCATAA
CCL2	ACAAGCAAACCCAAACTCCG	AACAGGGTGTCTGGGGAAAG
CCL3	TTCCGTCACCTGCTCAGAATC	GTGATGCAGAGAACTGGTTGC
CXCL8	CACTGCGCCAACACAGAAAT	TTCTCAGCCCTCTTCAAAAACTTC
CXCL10	AGTGGCATTCAAGGAGTACCT	CGTGGACAAAATTGGCTTGC
ICAM1	CCTTCCTCACCGTGTACTGG	AGCGTAGGGTAAGGTTCTTGC
IL6	AGCCACTCACCTCTTCAGAAC	GCCTCTTTGCTGCTTTCACAC
LPAR1	AATCGGGATACCATGATGAGTCTT	GGTGGCGCTCATTTCTTT
LPAR2	CGCTCAGCCTGGTCAAGACT	TTGCAGGACTCACAGCCTAAAC
LPAR3	AGGACACCCATGAAGCTAATGAA	TGCCATACATGTCCTCGT
LPAR4	TCTGTTTCCGCATGAAAATGAGA	TGCAGAGGGTGTCACCAAAAG
LPAR5	CACTTGGTGGTCTACAGCTTG	GCGTAGTAGGAGAGACGAACG
LPAR6	GATTGTTTGCACTGGCGTGT	AAGCAGGCTTCTGAGGCATT
